# Improving Association Studies and Genomic Predictions for Climbing Beans With Data From Bush Bean Populations

**DOI:** 10.3389/fpls.2022.830896

**Published:** 2022-04-25

**Authors:** Beat Keller, Daniel Ariza-Suarez, Ana Elisabeth Portilla-Benavides, Hector Fabio Buendia, Johan Steven Aparicio, Winnyfred Amongi, Julius Mbiu, Susan Nchimbi Msolla, Phillip Miklas, Timothy G. Porch, James Burridge, Clare Mukankusi, Bruno Studer, Bodo Raatz

**Affiliations:** ^1^Molecular Plant Breeding, Institute of Agricultural Sciences, ETH Zurich, Zurich, Switzerland; ^2^Bean Program, International Center for Tropical Agriculture (CIAT), Cali, Colombia; ^3^Bean Program, International Center for Tropical Agriculture (CIAT), Kampala, Uganda; ^4^Tanzania Agricultural Research Institute (TARI), Dodoma, Tanzania; ^5^Department of Crop Science and Horticulture, Sokoine University of Agriculture, Morogoro, Tanzania; ^6^Department of Agriculture, Agriculture Research Service (USDA-ARS), Prosser, WA, United States; ^7^Department of Agriculture, Agriculture Research Service (USDA-ARS), Tropical Agriculture Research Station, Mayaguez, PR, United States; ^8^Department of Plant Science, The Pennsylvania State University, University Park, PA, United States

**Keywords:** genome-wide association studies (GWAS), genomic selection, population structure, pleiotropy, growth habit, common bean (*Phaseolus vulgaris* L.), climbing and bush type bean

## Abstract

Common bean (*Phaseolus vulgaris* L.) has two major origins of domestication, Andean and Mesoamerican, which contribute to the high diversity of growth type, pod and seed characteristics. The climbing growth habit is associated with increased days to flowering (DF), seed iron concentration (SdFe), nitrogen fixation, and yield. However, breeding efforts in climbing beans have been limited and independent from bush type beans. To advance climbing bean breeding, we carried out genome-wide association studies and genomic predictions using 1,869 common bean lines belonging to five breeding panels representing both gene pools and all growth types. The phenotypic data were collected from 17 field trials and were complemented with 16 previously published trials. Overall, 38 significant marker-trait associations were identified for growth habit, 14 for DF, 13 for 100 seed weight, three for SdFe, and one for yield. Except for DF, the results suggest a common genetic basis for traits across all panels and growth types. Seven QTL associated with growth habits were confirmed from earlier studies and four plausible candidate genes for SdFe and 100 seed weight were newly identified. Furthermore, the genomic prediction accuracy for SdFe and yield in climbing beans improved up to 8.8% when bush-type bean lines were included in the training population. In conclusion, a large population from different gene pools and growth types across multiple breeding panels increased the power of genomic analyses and provides a solid and diverse germplasm base for genetic improvement of common bean.

## 1. Introduction

Common bean (*Phaseolus vulgaris* L.) was domesticated about 8,000 years ago in two geographic regions, resulting in the Andean and the Mesoamerican gene pools (Gepts et al., 1986; Bitocchi et al., 2013). Within the two gene pools, several groups, including climbing and bush type beans, were identified through genetic or phenotypic characterization (Gepts et al., 1986; Rodriguez et al., 2016). Seven domestication events for the common bean were discovered by investigating a genetic locus for flowering determinacy (Kwak et al., 2008, 2012). Flowering determinacy defines the first criterion for growth type: the determinate growth type forms a reproductive terminal bud, whereas the indeterminate growth types produce a vegetative one (Singh, 1981). The second criterion describes the bush vs. climbing growth habit. Both criteria were used to distinguish four growth types: type I (determinate bush), type II (indeterminate bush), type III (indeterminate semi-climber), and type IV (indeterminate climber) (Singh, 1981). Since growth type is associated with flowering determinacy, it also affects vegetative growth and the length of the crop cycle (González et al., 2016). In recent decades, major breeding efforts have been directed toward the erect growth habit of bush type beans since this habit enables a faster cultivation cycle without staking of the plants and a single, automated harvest (Teixeira et al., 1999; Ronner et al., 2018). The joint diversity of common bean growth types may offer new insights to improve not only climbing but also bush type beans.

Although largely neglected in breeding programs, climbing beans offer three main advantages over bush type beans: first, climbing beans reach higher yield per area, with up to 5 t ha^-1^ (Rosales-Serna et al., 2004; Checa et al., 2006; Barbosa et al., 2018). Second, they have a higher symbiotic nitrogen fixation capacity, with up to 92 kg of N fixed ha^-1^ (Graham, 1981; Bliss, 1993; Checa et al., 2006; Barbosa et al., 2018). Third, they achieve higher seed iron content (SdFe), with up to 10 mg/100 g (Blair et al., 2010; Blair, 2013; Petry et al., 2015; Mukamuhirwa and Rurangwa, 2018). Indeed, the production of climbing beans can be more profitable, and they are preferentially adopted in higher and/or drought prone regions by small holder farmers in Uganda and Rwanda (Ronner et al., 2018; Katungi et al., 2019). However, these advantages come with the cost of staking the plants and a longer vegetative period, partly due to the indeterminate growth type (White et al., 1992).

To shorten the cultivation cycle, Kornegay et al. (1992) suggested crossing type I and II bean lines with type IV lines to achieve an increase in yield while selecting against climbing ability. However, the climbing growth habit is tightly linked to plant development and productivity. The relation of days to flowering (DF), vegetative growth, and plant production was investigated in two Andean (type I) x Mesoamerican (type IV) recombinant inbred line populations (González et al., 2016). In those mixed climbing and bush type populations, the QTL containing the *PvTFL1y* flowering gene explained 32% of the variation for DF, 66% for vegetative growth (length of the main stem), and 19% for the rate of plant production, including traits such as yield and seed weight (González et al., 2016). In general, more days to physiological maturity (DPM) resulted in an increased yield of lines among and across the different growth types (White and Izquierdo, 1989; Keller et al., 2020). However, within growth type I and type II, DPM was not related to yield in near-isogenic lines (White et al., 1992). This suggests that the relationship between yield, DF, and DPM can be partially uncoupled.

The higher SdFe in climbing beans is promising to combat iron deficiency in human nutrition, which causes anemia, increases morbidity, and leads to economic losses (Boccio and Iyengar, 2003). About 30% of the global population suffers from anemia, especially women and children in developing countries (Black et al., 2008; Stein, 2010). Increasing SdFe in legumes is a possible avenue to improve nutritional quality in the human diet (Petry et al., 2015; Rehman et al., 2019). In the last years, a few biofortified lines with higher SdFe were successfully released (HarvestPlus, 2022). Iron biofortified beans showed higher phytic acid concentrations, which decreased the relative but not absolute iron absorption (Petry et al., 2014). However, the SdFe of climbing beans has not been investigated intensively and SdFe is negatively correlated with yield (Kelly and Bornowski, 2018). Such tradeoffs, including the longer DPM, which is associated with the climbing habit, need to be taken into account when improving climbing beans.

Efficient breeding for multiple traits requires detailed knowledge about the underlying genetic architecture of the target traits and their correlations with other key characteristics. The genetic architecture of traits can be investigated by genome-wide association studies (GWAS) and genomic prediction models (Crossa et al., 2017; Cortes et al., 2021). For common bean, GWAS have been carried out successfully and QTL for various traits were tagged with molecular markers (Miklas et al., 2006; Wu et al., 2020). Recently, genomic predictions were evaluated for agronomic traits in an elite Andean breeding panel (Keller et al., 2020). This study revealed the prediction abilities (PAs) for the genomic estimated breeding values (GEBV) based on genomic data only, as proposed by Meuwissen et al. (2001). This allows breeders to efficiently select superior lines before they enter field trials (Crossa et al., 2017). In general, PA is increased for more heritable traits and in lines, which are closely related to the training population (TP). In order to deal with different populations or breeding panels, multivariate models were suggested to account for population structure (Lehermeier et al., 2015). Following another approach, the TP can be optimized for genetic relatedness to improve the accuracy of the GEBV (Akdemir and Isidro-Sánchez, 2019; Sarinelli et al., 2019). These approaches have great potential to improve PAs, given the increasing availability of genotypic and phenotypic data (Spindel and McCouch, 2016). The efficient use of prediction models based on appropriate TPs and available data sets is a key factor in the development of new and adapted climbing bean cultivars.

In this study, comprehensive genetic analyses across all bean growth types and the two gene pools were carried out using 1,869 lines belonging to five breeding panels. We hypothesized that (*i*) climbing and bush type beans show, despite specific growth habit loci, overall a high genetic similarity, that (*ii*) combined analysis will increase the power of GWAS as well as the genomic predictions across all growth types and that (*iii*) predictions for climbing beans with an optimized TP including bush beans will outperform predictions which were based on the climbing bean growth types only. Hence, we evaluated whether breeding programs can supplement their trial data with already existing data to improve GWAS and genomics predictions. The overall objective was to provide molecular markers and genomic prediction models which can be used to speed up the selection of new bean lines across all growth types and gene pools.

## 2. Materials and Methods

### 2.1. Germplasm

The germplasm used in this study consisted of five bean breeding panels across all growth types: the newly composed climbing bean panel (VEC), the Andean diversity panel (ADP), the panel of progeny from two-way crosses between five Andean and five Mesoamerican parents (AxM), the Mesoamerican introgression panel (MIP), and the elite Andean breeding panel (VEF), whereas the latter four are bush type bean panels known from previous studies.

#### 2.1.1. Climbing Bean Panel

The VEC comprised climbing bean lines of growth types III and IV. The lines were selected for grain quality, commercial seed type, disease resistance, SdFe, seed zinc concentration (SdZn), and agronomic performance. They represent the genetic variation used in climbing bean breeding at the International Center for Tropical Agriculture (CIAT). The VEC was composed mainly of lines from the Andean gene pool, but it also included a line of the Mesoamerican gene pool (G2333) and a few lines of admixed origin. In total, the VEC was comprised of 290 lines including twelve breeding groups, four genebank accessions, and six cultivars (Supplementary Table 1).

Field trials including all VEC lines were conducted in Colombia, Uganda, and Tanzania to collect phenotypic data for this study.

#### 2.1.2. Bush Type Bean Panels

Four bush type breeding panels were used in this study: (*i*) the ADP consisting of 352 Andean bush cultivars and breeding lines from public and private breeding programs as described by Cichy et al. (2015). The ADP genotyping-by-sequencing (GBS) data was available *via* the ARS Feed-the-Future Grain Legumes Project (arsftfbean.uprm.edu/bean/?p=472); (*ii*) the AxM as described by Mayor Duran et al. (2016) and Mayor Duran (2016); (*iii*) the MIP consisting of Mesoamerican breeding lines with interspecific introgressions from *P. acutifolious, P. dumosus, and P. coccineus* in their pedigrees and their parental lines as described by Diaz et al. (2021); (*iv*) the VEF as described by Keller et al. (2020).

For the VEF, MIP, and AxM, phenotypic and genotypic data of 605, 217 and 200 lines were available, respectively. For the ADP, field trials were conducted in Mozambique, Tanzania, and in the United States to collect phenotypic data for this study.

### 2.2. Phenotyping

Agronomic traits were evaluated in the VEC and ADP as previously described by Keller et al. (2020). Briefly, DF represents the days from planting until 50% of the plants in the plot had at least one open flower. The seed yield per plot was normalized to a moisture content of 14% and extrapolated to yield per hectare. The weight of 100 seeds (100SdW) was measured separately. The growth type was assessed according to the four categories described by Singh (1981). The growth habit described climbing ability and differentiated only between bush (types I and II) and climbing types (type III and IV). Further traits such as DPM, pod harvest index (PHI), SdFe, SdZn, and canning quality were phenotyped only for the VEC. Analogous to DF, DPM represents the days until 50% of the pods in one plot had lost their green pigmentation. For the PHI, the seed dry weight of 20 pods at harvest was divided by the corresponding pod dry weight. The SdFe and SdZn were assessed on dried and ground seeds as described by Stangoulis and Sison (2008). The SdFe and SdZn content was then quantified by the X-ray fluorescence method using the X-Supreme 8000 instrument (Oxford Instruments, UK) (Guild et al., 2017). The canning quality was assessed by a trained sensory panel at Michigan State University as described by Cichy et al. (2014). Briefly, the beans were soaked in distilled water, canned at 100°C, sealed, and stored for 2 weeks. Upon opening, the canning quality was assessed by a trained consumer panel and expressed as an overall score from 1 (unacceptable appearance) to 5 (excellent appearance). The rated criteria included color, bean splitting, free starch clumps, and brine clarity after cooking (Cichy et al., 2014).

#### 2.2.1. Field Trials for Climbing Beans

The field trials for the VEC were carried out at five locations in three countries: Darién (3°53′31^′′^N 76°31′00^′′^W, altitude of 1,491 m a.s.l.), Palmira (3°30′03.0^′′^N 76°21′03.5^′′^W, altitude 965 m a.s.l.), and Popayán (2°25′39^′′^N 76°37′17^′′^W, altitude of 1,750 m a.s.l.) in Colombia; Kawanda (0°24′49^′′^N 32°31′59^′′^E, altitude of 1,190 m a.s.l.) in Uganda; and Kagera (1°24′56.5^′′^S 31°46′48.8^′′^E, altitude of 1,320 m a.s.l.) in Tanzania (Supplementary Table 2). Each line was arranged in an alpha lattice design with three replicates.

Regarding the field trials in Colombia, the experimental units consisted of one row with a row-to-row distance of 0.95 m and with seven seeds sown manually per meter row length. Row length per plot differed between locations with 2.5 m, 2.2 m, and 2.0 m used in Darién, Palmira, and Popayán, respectively (Supplementary Table 2). Climbing beans (mainly type IV) required a trellis to support the plant. Wooden poles of 3 m height were distributed in the field in squares of 5 x 5 m. Wires were spanned between the poles at a height of 2.3 m. Each bean plant climbed on a string hanging from the wire. Eight strings per plot were deployed. Plant protection was carried out when needed using good agricultural practices.

The soil type in Darién and Popayán was an Inceptisol (Typic Dystrandept) with about 70 g/kg and 140 g/kg of soil organic matter, respectively, and a pH of around 5 (Barbosa et al., 2018). The soil type in Palmira was Mollisol with a pH between 7.0 and 7.5.

In Uganda and Tanzania, the plots were planted in single 3.0 m rows with about 30 seeds planted per row and at 0.6 m between the rows (Supplementary Table 2). Granular N:P:K (nitrogen, *P*_2_0_5_ and *K*_2_*O*) fertilizer was manually applied at 125 kg/ha. Trials were manually weeded two times. Climbing bean plants were staked with 2 to 3 m long wooden poles. Five poles were used per plot.

#### 2.2.2. Field Trials for Bush Beans

The field trials for the ADP were carried out at five locations in three countries: Arusha (3°21′41^′′^S 36°37′34^′′^E, altitude of 1,387 m a.s.l.), Morogoro (6°51′14.2^′′^S 37°39′27.6^′′^E, altitude of 526 m a.s.l.), and Mbeya (8°54′52^′′^S 33°31′05^′′^E, altitude of 1,780 m a.s.l.) in Tanzania; Chokwe (24°30′04^′′^S 33°00′09^′′^E, altitude of 35 m a.s.l.) in Mozambique; and Wilcox (32°01′44^′′^N 109°41′27^′′^W, altitude of 1,321 m a.s.l.) in the United States (Supplementary Table 3).

The trials were conducted in two to three replications in a randomized complete block design (Supplementary Table 3). The plot size differed between trials with lengths of 5–8 m, 1–0.5 m spacing between rows, and 1–4 rows. The plot sizes, soil types, and growth conditions of each trial are described in Supplementary Table 3. Irrigated trials were watered about two times a month and drought trials were rain fed. The growing season of one trial (MZCH15D_heat) coincided with the high temperature season at the site. The trials in Tanzania and in the United States were not fertilized. In the trials in Mozambique, single superphosphate and ammonium sulfate were applied at about 30 kg P/ha and 6.3 kg N/ha, respectively.

#### 2.2.3. Available Phenotypic Data From Previous Studies

Of the 290 VEC lines, 43 were phenotypically characterized in two trials in a previous study (Barbosa et al., 2018). Phenotypic data were also publicly available for the AxM (Mayor Duran, 2016), the MIP (Diaz et al., 2021), and the VEF (Keller et al., 2020) from ten, one and three trials, respectively (Supplementary Table 4).

### 2.3. Genotyping

GBS was conducted as previously described by Nay et al. (2019). Briefly, DNA was extracted from leaf tissue and digested with the ApeKI restriction enzyme as described in Elshire et al. (2011). The sequencing was performed on the Illumina HiSeq 2500 platform at the HudsonAlpha Genome Sequencing Center (Huntsville, AL, United States). The sequence reads were demultiplexed using NGSEP (v3.3.0) (Tello et al., 2019), trimmed using Trimmomatic (v0.36) (Bolger et al., 2014), and aligned to the reference genome of *P. vulgaris* G19833 v2.1 (Schmutz et al., 2014) using Bowtie2 (v2.2.30) (Langmead and Salzberg, 2012). The variant calling was carried out using NGSEP, filtering SNPs with a genotype quality below 40, minor allele frequency (MAF) below 0.05, and removing SNPs with less than 60% of genotype calls, which yielded a matrix with 20% of missing data. The imputation of the missing data was performed with Beagle v.5.0 (Browning et al., 2018) using 100 as an effective population size and using the genetic map reported by Diaz et al. (2019). The SNP calling was carried out once on the VEC separately and once on all five panels together. All VEC lines were genotyped together with 55 additional climbing lines from a previous study (Barbosa et al., 2018).

### 2.4. Data Analysis

#### 2.4.1. Phenotypic Data

Best linear unbiased estimators (BLUEs) were extracted from phenotypic data in two stages. In the first stage, the field data were corrected for spatial effects using the SpATS R package, setting row and column as random effects (Rodríguez-Álvarez et al., 2018). The number of plants harvested was binned (binwidth = 5) and added as a random effect in the spatial analysis. The plots with residuals bigger than ±3 times the SD were treated as outliers and removed iteratively as described in Keller et al. (2020). The BLUEs were extracted for each line in each trial from the SpATS model (first-stage BLUEs) from all the data sets, except for the ADP. In the ADP lines, the first-stage BLUEs were extracted using replicate (block) as a factor for the fixed effects since the row and column information was not available for these randomized complete block design trials. In the second stage, second-stage BLUEs for each line (*L*_*i*_) were calculated across all trials using the following model:


(1) $$\begin{array}{l} y_{ij}=L_{i}+E_{j}+\varepsilon_{ij} \end{array}$$

where *y*_*ij*_ is the first-stage BLUE of the *i*^*th*^ line in the *j*^*th*^ trial, *L*_*i*_ is the fixed effect for each line, *E*_*j*_ is the fixed effect for each trial, and ε_*ij*_ the error term. The inverse of the squared standard error (SE) of the mean was used as a weight, i.e., ε~*N*(0, *R*); $R={⊕}_{j=0}^{n}R_{j}$ where $R_{j}=diag\left[{\left(SE_{ij}\right)}^{2}\right]$, and (*SE*_*ij*_) is the standard error of a mean of the *i*^*th*^ line in the *j*^*th*^ trial (Möhring and Piepho, 2009). In addition, BLUEs for yield were scaled (mean = 0, SD=1 resulting in Yd_scaled) for each panel to compare relative differences between the lines beyond panel and growth type. Kernel density estimates of the BLUEs were calculated using a Gaussian kernel with 1/30 bandwidth of the data range. The estimates were drawn as a smoothed histogram with the integral equal to one using the ggplot R package (Wickham, 2016). The significance of the genotype-by-environment interaction (GxE) was tested by comparing model 1 with and without an interaction term *EL*_*ij*_ between *j*^*th*^ trial and *i*^*th*^ line using the likelihood-ratio test. The test statistic was compared with a chi-square value with one degree of freedom and the *p*-value was adjusted following the work by Self and Liang (1987).

#### 2.4.2. Linkage Disequilibrium and Population Structure

Pairwise measures of linkage disequilibrium (LD) were calculated for each population in sliding windows of 100 markers. The LD measures were corrected for kinship in the population ($r_{V}^{2}$) as implemented in the R package LDcorSV (v1.3.2) (Mangin et al., 2012). The LD decay was estimated regressing the pairwise $r_{V}^{2}$ values on the physical distance of their markers using the locally estimated scatterplot smoothing implemented in the R function “loess” (v4.1.0), with a span value of 0.5.

The phylogenetic tree was constructed using the GGTREE R package on the hierarchically clustered SNP matrix (Yu et al., 2017). The population structure was assessed on the SNP matrix using principal components analysis (PCA) implemented in the FactoMineR R package (Lê et al., 2008). The correlation of the supplemental phenotypic traits (second-stage BLUEs) with the principal components (PCs) of the SNP matrix was calculated using the same FactoMineR package (Lê et al., 2008).

#### 2.4.3. Genome-Wide Association Studies

To carry out GWAS, the Bayesian-information and Linkage-disequilibrium Iteratively Nested Keyway (BLINK) algorithm implemented in GAPIT was used (Huang et al., 2019). The first five PCs were used to correct for population structure. The imputed SNP matrix was used for GWAS. Identified QTL tagged by SNP markers were labeled with “Trait_Chr_Postion” as QTL ID, whereas yield was abbreviated with Yd and growth habit with GH. The SNP position derived from the reference genome G19833 v2.1 (Schmutz et al., 2014) was in Mbp rounded to two digits.

The network of significant marker-trait associations was visualized similarly as suggested in Fang et al. (2017) using the ggnetwork R package (Briatte et al., 2020). The distance between the connected nodes represents the LD between the two SNPs calculated as the squared Pearson correlation coefficient. The SNPs were connected when LD >0.25. The haplotypes associated with one trait were assembled using the identified SNPs in the indicated region for that trait. In case there were less than six SNPs selected, these SNPs were directly assembled to haplotypes. When more than five SNPs were selected, groups of haplotypes were constructed by hierarchical clustering of all lines based on those SNPs using the stats R package. The optimal number of clusters was determined using the average silhouette width implemented in the factoextra R package (Kassambara and Mundt, 2020).

### 2.5. Genomic Prediction

The GEBV were estimated using Bayesian generalized linear regression (BGLR) implemented in the BGLR R package (Pérez and de los Campos, 2014). For the factorial models, the BGLR extension for the Multiple-Trait Model (MTM) was used (http://quantgen.github.io/MTM/vignette.html). The Gibbs sampler ran with 20,000 iterations of which the first 10,000 were burned-in and the remaining were thinned by factor 5. Three different model approaches were tested.

#### 2.5.1. Genotype Model Among and Across Trials

For each trait, the phenotypes adjusted per trial (*y*_*ij*_) were modeled as the sum of the GEBV for each line (*g*_*i*_) estimated based on SNP marker information, i.e., the additive relationship matrix (K), a fixed effect for the trial (*E*_*j*_), and an error term $\varepsilon_{ij}\sim N\left(0,\sigma_{\varepsilon }^{2}\right)$. The following linear model was used:


(2) $$\begin{array}{l} y_{ij}=g_{i}+E_{j}+\varepsilon_{ij} \end{array}$$

with $g\sim N\left(0,K\sigma_{g}^{2}\right)$ and K was calculated as the normalized cross product of the SNP matrix using the rrBLUP package (Endelman, 2011; Endelman and Jannink, 2012). The *y*_*ij*_ were calculated either among all trials (using first-stage BLUEs), for all trials separately (using first-stage BLUEs), or across all trials (using second-stage BLUEs). In the case of the genotype model using all trials separately, j represents always the same environment. The same applied to the genotype model when using second-stage BLUEs across all trials.

#### 2.5.2. GxE Model

In the GxE model, the GEBV for each trait were estimated for each environment (i.e., location) by adding an effect for the interaction between the *j*^*th*^ environment and *i*^*th*^ GEBV (*gE*_*ij*_) to the model (2). This resulted in the following model:


(3) $$\begin{array}{l} y_{ij}=g_{i}+E_{j}+gE_{ij}+\varepsilon_{ij} \end{array}$$

with $gE\sim N\left(0,I⊗K\sigma_{gE}^{2}\right)$, where I is the identity matrix for the environments and ⊗ denotes the Kronecker product. This means no correlations between environments were considered.

#### 2.5.3. Factor Analysis Model

In the factor analysis (FA) model, the GEBV for each line in each environment (*g*_*ij*_) were estimated for each trait using SNP marker information and the covariance of the phenotypes (*y*_*ij*_) between the trials. The following equation was used:


(4) $$\begin{array}{l} y_{ij}=g_{ij}+\varepsilon_{ij} \end{array}$$

with $g\sim N_{j}\left(0,G⊗K\sigma_{g}^{2}\right)$, where G represents a covariance matrix of phenotypes between trials calculated as *G* = *BB*^*T*^+Ψ, where *B* is a matrix of loadings (regressions of the original random effects into common factors) and Ψ is a diagonal matrix whose non-null entries give the variances of factors that are trait-specific. The model residuals were assumed to follow a multivariate normal distribution ε~*N*_*j*_(0, *R*_ε_⊗*I*_*n*_), where *R*_ε_ is a covariance matrix of model residuals and *I*_*n*_ represents an *n*-dimensional identity matrix, where *n* is the number of phenotypes per environment. Three common factors were selected.

#### 2.5.4. Training Population Optimization

For each VEC line in the validation set, the 50 closest related lines from all five panels were added to the TP (TP optimized). Genetic relationships were calculated as the cophenetic distances of the hierarchically clustered lines based on the SNP matrix, i.e., as the height of the dendrogram where the two branches including the considered lines join each other. This means that the TP could consist of between 50 lines (in case the lines to be tested would have all the same 50 closest relatives) and 2,500 lines (if they would not have the same closest relatives). The number of related lines (50) was chosen arbitrarily but seemed reasonable for a panel of 1,500 lines with some population structure. The optimized TP was compared to a TP containing only VEC lines (TP VEC) and a TP containing all lines from all five panels (TP extended).

#### 2.5.5. Cross-Validation

The cross-validation was done 100 times, randomly splitting the dataset into training and validation set, i.e., for each cross-validation step, 70% of the lines were selected for the training and 30% for the validation set. The lines in the training and validation sets were referred to as TP and new lines, respectively. The Pearson correlation coefficient (*r*) between predicted and observed values was calculated at each cross-validation step to assess the PA. The prediction accuracy (PAcc) is defined as the quotient of PA and the square root of heritability.

#### 2.5.6. Genomic BLUPs Without Cross-Validation

The genomic *r* is defined as the Pearson correlation coefficient of modeled vs. observed values when all lines were used in the TP to fit the model. The genomic *r* was derived from the predicted genomic BLUPs without cross-validation.

## 3. Results

The traits DF, 100SdW, SdFe, and yield were analyzed in 27, 28, 6, and 33 field trials, respectively, using a total of 1,869 lines belonging to five different breeding panels (Supplementary Figure 1). For this study, six and eleven field trials were newly evaluated, adding data for the VEC and ADP, respectively. The number of evaluated lines per trial was 290 for the VEC (Supplementary Table 2) and ranged between 41 and 268 for the ADP (Supplementary Table 3).

### 3.1. Phenotypes of Climbing Beans

**Figure 1:**
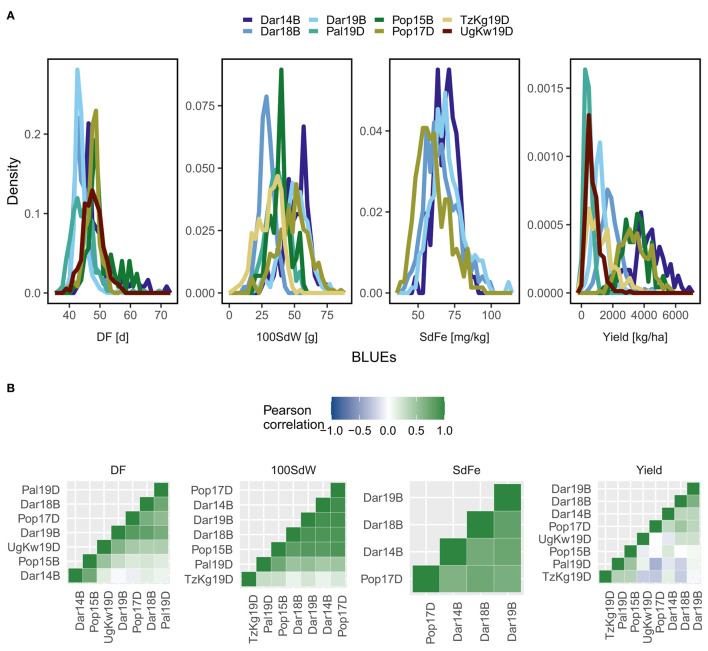
Phenotypes of the climbing bean panel (VEC). **(A)** Density diagrams for days to flowering (DF), 100 seed weight (100SdW), seed iron concentration (SdFe), and seed yield of up to 290 VEC lines in eight trials are shown. Best linear unbiased predictors (BLUEs) were calculated from each trial corrected for spatial effects in the field. **(B)** Pearson correlation coefficients were calculated across all trials for each trait based on the BLUEs. Trials were abbreviated based on the location Darién (Dar), Palmira (Pal), Popayán (Pop) in Colombia, Kagera in Tanzania (TzKg), or Kawanda in Uganda (UgKw), the year and the planting season (sequentially A to D). For a detailed description of each trial refer to Supplementary Table 2.

The lines of the VEC showed different phenotypic distributions for the traits DF, 100SdW, SdFe, and yield among all field trials (Figure 1A). Especially, strong environmental effects were observed for yield. The trials carried out in the lowlands and in the warmer climates (Palmira, Kawanda, and Kagera) had less yield compared to the remaining trials in the high-altitude locations Darién and Popayán. Phenotypic variation among and across trials was also observed for additional traits, including important breeding targets such as canning quality and PHI (Supplementary Figure 2). The phenotypic correlations between trials were evaluated using the first-stage BLUEs: positive correlations were revealed across the trials for DF, 100SdW, and SdFe, whereas yield of the Pal19D and TzKg19D trials were mainly negatively correlated to the other trials (Figure 1B). The likelihood-ratio test confirmed significant GxE (*p* value <0.001) for all traits, especially for yield, where the variance component for GxE was higher than for the lines (Supplementary Table 5).

Across all trials, the correlations between traits were evaluated using the second-stage BLUEs. Positive correlations were revealed between the traits SdFe, SdZn, DPM, and DF (Supplementary Figure 3). However, the correlations were negative between yield, nitrogen use efficiency, and 100SdW. Additionally, PHI was negatively correlated to SdFe and SdZn. Since yield showed strong GxE, the correlations to other traits differed across trials, e.g., the yield was negatively correlated with DF in the Pop15B and Pal19D trials but positively correlated with DF in the remaining trials (Supplementary Figure 4). In summary, strong GxE was observed for yield, while the remaining traits showed moderate GxE among the different environments and trials.

### 3.2. Comparing Phenotypes of the Five Breeding Panels

**Figure 2:**
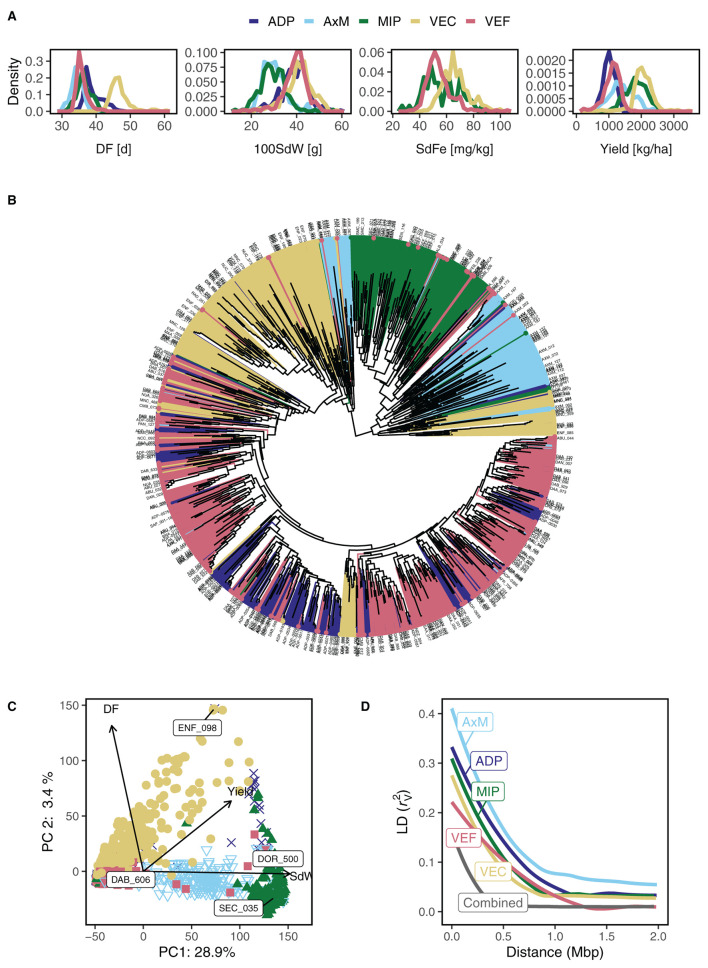
Phenotypic and genotypic characterization of five common bean breeding panels including lines with bush and climbing growth habit originating from the Andean and Mesoamerican gene pools. **(A)** Density diagrams of best linear unbiased estimators among five breeding bean panels are shown for days to flowering (DF), 100 seed weight (100SdW), seed iron concentration (SdFe), and seed yield. **(B)** Dendrogram of 1,869 lines characterized by 14,913 SNPs shows the hierarchical relationships between lines and panels [following the same color code for panels as in **(A)**]. **(C)** Principal components (PC) 1 and 2 visualize the genetic similarity across all five breeding panels. The arrows show quantitative supplementary phenotypic traits. Their cosines indicate the correlation with PC axes and their length approximate the SD of the variable. The extreme lines on the PC 1 axis are labeled. **(D)** Linkage disequilibrium (LD) decay is shown for all panels separately and combined. The LD was calculated in sliding windows of 100 markers and corrected for kinship in the population ($r_{V}^{2}$).

The phenotypic distribution of DF, 100SdW, SdFe, and yield were compared among all five panels across all trials (Figure 2A). The climbing beans of the VEC showed on average 28%, 21%, and 67% higher DF, SdFe, and yield than the bush type panels, respectively. The trait 100SdW depended primarily on the gene pools, showing lower values in the Mesoamerican MIP and the AxM, consisting of inter gene pool crosses. The observation in the VEC, that SdFe correlated negatively and DF and 100SdW positively to yield, was also true in the four bush bean panels (Supplementary Figure 4).

### 3.3. Structure and Diversity of the Five Breeding Panels

In the joint analysis of all five panels, 14,913 SNP markers distributed over the whole genome were kept from the raw 169,087 SNPs after filtering for genotype quality calls, MAF, and missingness. The dendrogram of the 1,869 lines showed grouping into Mesoamerican (represented by the MIP) and Andean origin, whereas the AxM and part of the VEC formed an admixture branch (Figure 2B). These genetic groups were also visible in the PCA analysis of all lines: the first PC clearly grouped the lines according to their Andean and Mesoamerican origin spreading the admixed AxM lines in-between (Figure 2C). In agreement, the first PC was highly correlated with 100SdW (*r* = −0.67) which differentiated the two gene pools (Figures 2A,C). The second PC explained variation for growth type, separating mostly the VEC lines from the others, and was correlated to DF (*r* = 0.58, Figure 2C). The first and second PC explained 28.9 and 3.4% of the genetic variance, respectively. The third PC, explaining 2.4% of the variance, captured variation mainly between the AxM and MIP, whereas the fourth and fifth PC showed the smallest variation for the VEF (Supplementary Figure 5). Finally, the sixth PC showed no clear pattern among the panels. Therefore, five first PCs were included as fixed effects for GWAS. The LD decay observed for all the breeding panels was faster for the combined panel than for the separate panels, enabling higher detection power for GWAS (Figure 2D). In general, the genetic diversity was bigger between the two gene pools than between the growth types.

### 3.4. Genome-Wide Association Studies Across All Panels

**Figure 3:**
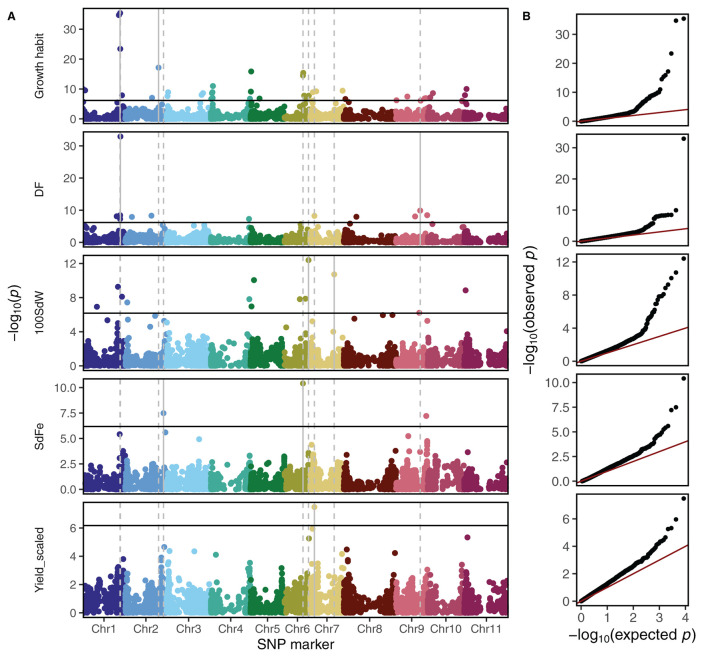
Genome-wide association studies among five breeding panels differing in growth habits. **(A)** The Manhattan plots show the genetic associations with climbing growth habit, days to flowering (DF), 100 seed weight (100SdW), seed iron concentration (SdFe), and seed yield. Seed yield was scaled among panels to allow comparison between them. The horizontal black lines show the Bonferroni corrected significance threshold at the 1% level. The vertical lines indicate the position of the two most significant markers for each trait. **(B)** Quantile distribution plots show the deviation of expected to observed *p*-values of SNP to trait associations for each trait.

Carrying out GWAS using the second-stage BLUEs across all trials and breeding panels, a total of 69 significant marker-trait associations were identified below the 1% Bonferroni corrected significance level (Figure 3A and Table 1). The observed *p*-value distribution showed a clear deviation of the identified significant SNPs from the expected uniform distribution if no genetic linkage was present (Figure 3B). A *p*-value inflation was visible mainly in growth habit and 100 SdW. Most striking was the region at around 45 Mbp on Chr 1 associated with significant effects for growth habit, DF, DPM, and 100 SdW (Supplementary Figure 6). Furthermore, different QTL for SdFe and scaled yield across all panels were identified.

**Table 1:** Significant marker-trait associations across five bean breeding panels below the 1% significance level according to genome-wide association studies.

**QTL ID**	**Trait**	**Chr**	**Pos**	***p*-value**	**Marker**	**MAF**
GH_1_0.53	Growth habit	1	526025	1.18E-10	Pv2.1_01_526025_A/T	0.24
GH_1_2.85	Growth habit	1	2850955	2.78E-10	Pv2.1_01_2850955 C/G	0.38
GH_1_43.71	Growth habit	1	43707108	1.93E-35	Pv2.1_01_43707108 A/G	0.41
GH_1_45.04	Growth habit	1	45044047	3.90E-36	Pv2.1_01_45044047 C/T	0.13
GH_1_45.37	Growth habit	1	45374662	4.01E-24	Pv2.1_01_45374662 T/C	0.08
GH_1_47.44	Growth habit	1	47439968	1.23E-08	Pv2.1_01_47439968 G/C	0.16
GH_2_32.21	Growth habit	2	32208182	9.73E-08	Pv2.1_02_32208182 C/G	0.32
GH_2_40.05	Growth habit	2	40045968	6.88E-18	Pv2.1_02_40045968 C/A	0.05
GH_3_1.29	Growth habit	3	1289075	2.35E-08	Pv2.1_03_1289075 T/A	0.45
GH_3_1.92	Growth habit	3	1921492	1.22E-09	Pv2.1_03_1921492 A/G	0.03
GH_3_42.49	Growth habit	3	42488118	9.24E-09	Pv2.1_03_42488118 G/C	0.3
GH_3_44.06	Growth habit	3	44056005	2.62E-09	Pv2.1_03_44056005 A/T	0.12
GH_4_0.45	Growth habit	4	448766	2.45E-07	Pv2.1_04_448766 A/T	0.03
GH_4_1.42	Growth habit	4	1421295	2.52E-09	Pv2.1_04_1421295 A/G	0.47
GH_4_1.92	Growth habit	4	1919859	1.29E-09	Pv2.1_04_1919859 A/G	0.26
GH_4_2.16	Growth habit	4	2164168	9.82E-08	Pv2.1_04_2164168 T/C	0.08
GH_4_2.56	Growth habit	4	2559941	1.04E-11	Pv2.1_04_2559941 A/T	0.25
GH_4_47.17	Growth habit	4	47174835	1.95E-07	Pv2.1_04_47174835 T/A	0.26
GH_5_0.39	Growth habit	5	394641	7.38E-10	Pv2.1_05_394641 T/G	0.13
GH_5_0.74	Growth habit	5	739798	1.33E-16	Pv2.1_05_739798 G/A	0.28
GH_5_10.7	Growth habit	5	10696009	1.63E-07	Pv2.1_05_10696009 G/C	0.02
GH_6_22.3	Growth habit	6	22301003	3.03E-15	Pv2.1_06_22301003 A/G	0.03
GH_6_22.51	Growth habit	6	22508433	3.94E-16	Pv2.1_06_22508433 T/G	0.16
GH_6_23.87	Growth habit	6	23868436	1.66E-08	Pv2.1_06_23868436 G/C	0.37
GH_6_26.05	Growth habit	6	26054074	2.23E-07	Pv2.1_06_26054074 C/T	0.1
GH_6_29.43	Growth habit	6	29429040	1.53E-08	Pv2.1_06_29429040 G/A	0.02
GH_7_3.05	Growth habit	7	3047903	1.18E-09	Pv2.1_07_3047903 G/A	0.28
GH_7_7.15	Growth habit	7	7150019	6.01E-10	Pv2.1_07_7150019 C/T	0.1
GH_7_38.99	Growth habit	7	38987037	3.73E-10	Pv2.1_07_38987037 A/G	0.42
GH_8_2.11	Growth habit	8	2107245	2.42E-07	Pv2.1_08_2107245 C/T	0.23
GH_9_0.86	Growth habit	9	860918	6.16E-07	Pv2.1_09_860918 G/A	0.17
GH_9_13.92	Growth habit	9	13924731	3.14E-08	Pv2.1_09_13924731 T/C	0.49
GH_9_34.74	Growth habit	9	34742076	1.48E-07	Pv2.1_09_34742076 A/G	0.23
GH_9_36.11	Growth habit	9	36110952	1.03E-07	Pv2.1_09_36110952 A/T	0.01
GH_10_3.19	Growth habit	10	3191949	6.26E-08	Pv2.1_10_3191949 G/T	0.21
GH_10_6.5	Growth habit	10	6495216	2.67E-09	Pv2.1_10_6495216 G/T	0.27
GH_11_1.01	Growth habit	11	1010422	1.14E-08	Pv2.1_11_1010422 T/C	0.49
GH_11_2.78	Growth habit	11	2775768	9.58E-11	Pv2.1_11_2775768 T/G	0.16
DF_1_41.08	DF	1	41082526	7.72E-09	Pv2.1_01_41082526 G/A	0.22
DF_1_44.6	DF	1	44604072	1.88E-08	Pv2.1_01_44604072 A/C	0.35
DF_1_44.93	DF	1	44927394	5.64E-09	Pv2.1_01_44927394 C/T	0.25
DF_1_45.04	DF	1	45044047	5.11E-08	Pv2.1_01_45044047 C/T	0.13
DF_1_45.23	DF	1	45233651	3.51E-09	Pv2.1_01_45233651 A/G	0.08
DF_1_45.47	DF	1	45469012	1.21E-33	Pv2.1_01_45469012 G/A	0.1
DF_2_7.9	DF	2	7900892	1.22E-08	Pv2.1_02_7900892 A/C	0.26
DF_2_31.53	DF	2	31525478	4.99E-09	Pv2.1_02_31525478 A/C	0.16
DF_4_45.98	DF	4	45975326	5.51E-08	Pv2.1_04_45975326 T/C	0.38
DF_7_4.82	DF	7	4824001	5.29E-09	Pv2.1_07_4824001 C/G	0.26
DF_8_15.48	DF	8	15481886	1.12E-08	Pv2.1_08_15481886 T/A	0.01
DF_9_23.12	DF	9	23116201	9.41E-09	Pv2.1_09_23116201 C/T	0.28
DF_9_29.38	DF	9	29378513	1.25E-10	Pv2.1_09_29378513 C/T	0.07
DF_9_37.83	DF	9	37825106	3.55E-09	Pv2.1_09_37825106 T/G	0.21
SdW_1_17.16	100SdW	1	17164005	1.19E-07	Pv2.1_01_17164005 A/G	0.16
SdW_1_42.52	100SdW	1	42515535	5.49E-10	Pv2.1_01_42515535 A/G	0.07
SdW_1_47.26	100SdW	1	47260087	7.66E-09	Pv2.1_01_47260087 A/G	0.28
SdW_2_2.23	100SdW	2	2234763	3.64E-08	Pv2.1_02_2234763 T/G	0.45
SdW_4_46.54	100SdW	4	46540184	1.51E-08	Pv2.1_04_46540184 C/G	0.36
SdW_5_1.06	100SdW	5	1062441	1.09E-07	Pv2.1_05_1062441 C/A	0.44
SdW_5_4.07	100SdW	5	4065359	8.94E-11	Pv2.1_05_4065359 C/T	0.28
SdW_6_18.46	100SdW	6	18456447	1.48E-08	Pv2.1_06_18456447 G/A	0.2
SdW_6_25.25	100SdW	6	25248245	1.34E-08	Pv2.1_06_25248245 G/C	0.21
SdW_6_28.9	100SdW	6	28898246	3.91E-13	Pv2.1_06_28898246 G/A	0.16
SdW_7_28.77	100SdW	7	28765036	1.85E-11	Pv2.1_07_28765036 C/T	0.02
SdW_9_28.29	100SdW	9	28287352	6.16E-07	Pv2.1_09_28287352 G/A	0.12
SdW_11_1.55	100SdW	11	1547189	1.39E-09	Pv2.1_11_1547189 C/T	0.12
SdFe_2_46.07	SdFe	2	46068228	3.20E-08	Pv2.1_02_46068228 G/C	0.19
SdFe_6_22.37	SdFe	6	22365971	3.95E-11	Pv2.1_06_22365971 A/C	0.32
SdFe_9_36 8	SdFe	9	36804490	6.26E-08	Pv2.1_09_36804490 C/T	0.16
Yd_7_4.86	Yield scaled	7	4856975	3.28E-08	Pv2.1_07_4856975 C/T	0.23

*The QTL ID, trait, chromosome (Chr), physical position (Pos) in bp, association strength (p value), name of the marker, and minor allele frequency (MAF) of the significantly associated SNPs is reported. The panels included bush and climbing growth habits and were evaluated for days to flowering (DF), 100 seed weight (100SdW), seed iron concentration (SdFe), and seed yield (Yield_scaled, scaled for each panel)*.

#### 3.4.1. Growth Habit and Pleiotropy

For growth habit, 38 significant marker-trait associations were detected (Table 1). These QTL determined growth habit, e.g., bush (type I and II) from climbing beans (type III and IV) or they distinguished growth determinacy, e.g., separated determinate types I from indeterminate type II, III, and IV (Supplementary Figure 7A). The SNPs associated with growth habits on Chr 1, 3, and 6 (GH_1_43.71, GH_3_1.29, and GH_6_23.87) differentiated mainly the determinate growth type I from the other three types. In contrast, two QTL on Chr 4 (GH_4_1.42 and GH_4_40.05) and on QTL on Chr 5 (GH_5_0.74) differentiated bush from climbing types. The minor SNP variant on Chr 2 (GH_2_40.05) was the only one exclusively associated with the two climbing growth types (type III and IV). However, this association is to interpret cautiously since this SNP variant was rare (MAF = 0.05).

The region at the end of Chr 1 showed significant pleiotropic effects on different traits (Supplementary Figure 6). In that region, significant SNPs were identified not only for growth habits but also for DF, DPM, and 100SdW. Additionally, the significant SNPs for growth habit on Chr 3 and 6 showed a tendency toward pleiotropic effects as observed for the QTL on Chr 1 (Supplementary Figure 7B). Interestingly, the QTL on Chr 4 (GH_4_1.42 and GH_4_40.05) showed again a different pattern than the others: these SNPs increased 100SdW with increasing DF while, surprisingly, yield (scaled across populations) decreased. In summary, the 38 SNPs significantly associated with growth habit determined the four different growth types in different proportions, whereas only a few of these SNPs expressed pleiotropic effects.

#### 3.4.2. SNP Effects Among Breeding Panels

**Figure 4:**
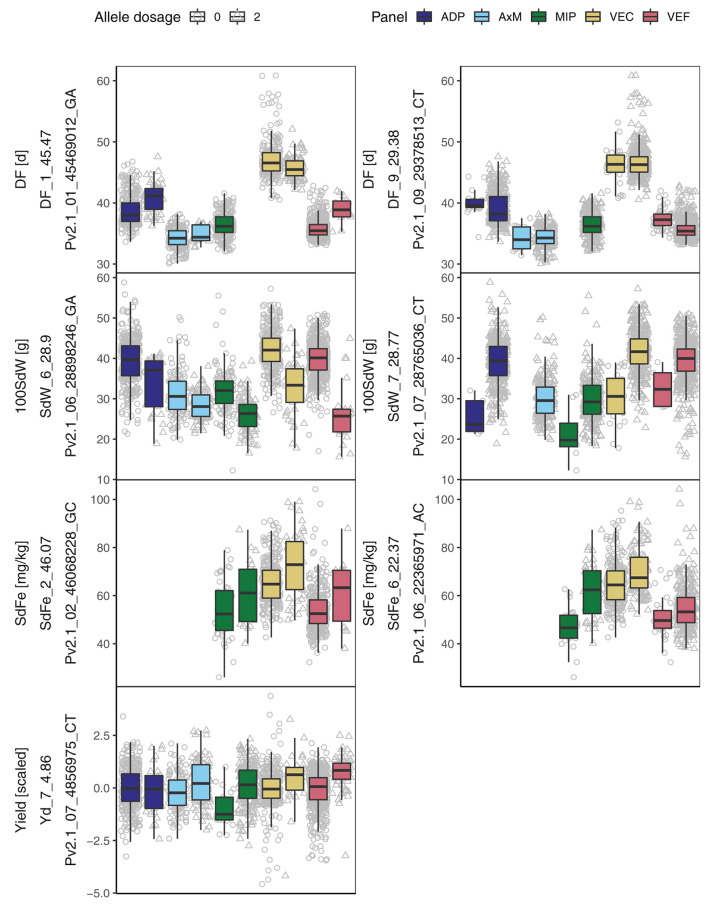
Boxplots for allele dosage effect (0 or 2 alternative alleles) of the most significant SNPs associated with days to flowering (DF), 100 seed weight (100SdW), seed iron concentration (SdFe), and seed yield for five breeding panels. The panels included lines with bush and climbing growth habits originating from the Andean and Mesoamerican gene pools. The trait, the name of the associated marker and the QTL ID is given.

**Figure 5:**
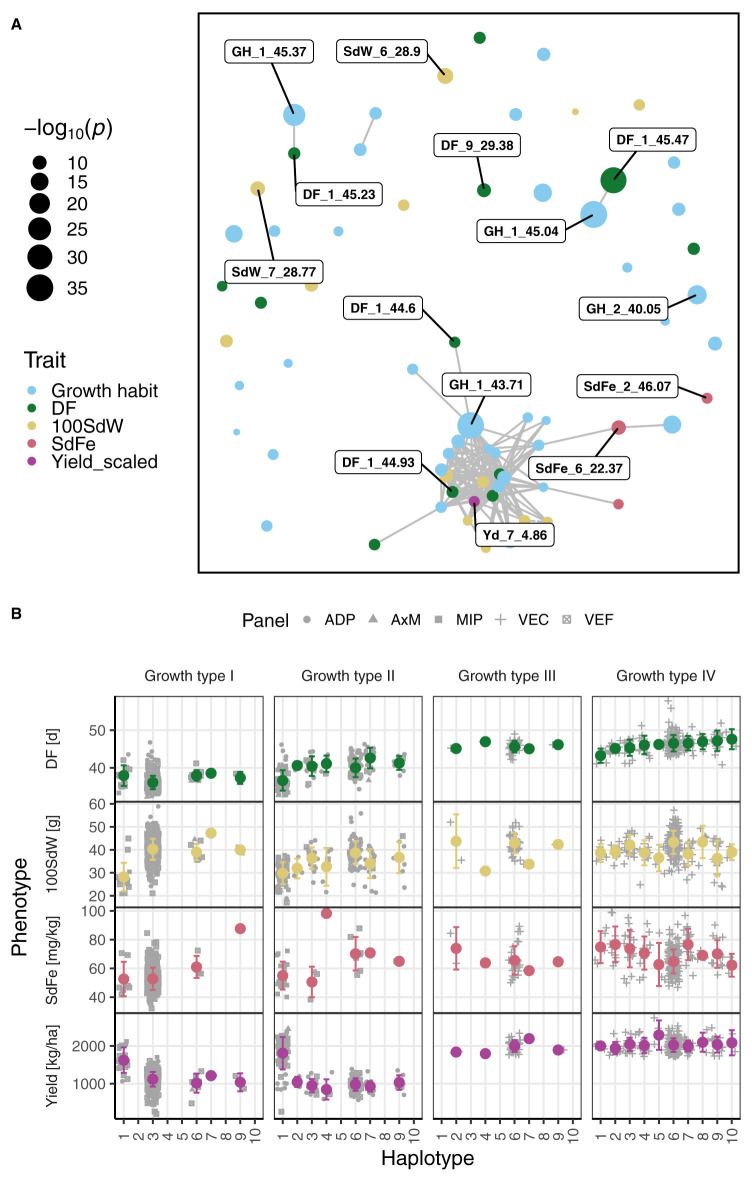
Significant marker-trait associations were analyzed for growth habit, days to flowering (DF), 100 seed weight (100SdW), seed iron concentration (SdFe), and seed yield scaled among the five breeding panels (Yield_scaled). **(A)** A network of the SNPs significantly (below the 1% significance level) associated with each of the four traits forming clusters according to their linkage disequilibrium is shown. Each dot represents a significant SNP and its size the associated −*log*_10_ *p*-value. The SNPs on Chr 1 between 43.71 and 45.47 as well as the two most significant SNPs per trait are labeled with the QTL ID. **(B)** Haplotypes including all SNPs between 44.60 and 45.47 Mbp on chromosome 1 were constructed using hierarchical clustering. The averaged SNP effects of the haplotypes were evaluated in all traits among all growth types, i.e., growth type I (determinate bush type), type II (indeterminate bush), type III (determinate climber), and type IV (indeterminate climber). The error bars show the SD.

In general, the significant marker-trait associations identified in the whole population of 1,869 lines showed effects also in the panels separately (Figure 4). An exception was the QTL DF_9_29.38 which revealed significant associations only in the VEF and ADP, the two Andean panels. An interesting breeding target for yield is Yd_7_4.86, expressing a clear effect in all five panels. This QTL was physically linked to DF_7_4.82 and in LD with several other QTL on different chromosomes (Figure 5A). Finally, two QTL for SdFe (SdFe_2_46.07 and SdFe_6_22.37) showed an effect in all three panels which had data for SdFe (Figure 4). Except for DF, major QTL for 100SdW, SdFe, and yield were identified showing effects in all five breeding panels.

#### 3.4.3. Haplotype Effects Across and Among Growth Types

For traits with complex genetic architecture, single SNPs poorly explain the variance caused by the associated genetic region. Therefore, haplotypes were constructed on Chr 1 between 43.71 and 45.47 Mbp for SNPs significantly associated either with DF or growth type. The haplotypes for DF on Chr 1 explained 44.6% of the variance for DF across all growth types (Supplementary Figure 8A). In contrast, the best SNP for DF on Chr 1 explained only 8% of the variance. The haplotypes for growth type on Chr 1 differentiated type I from the other types (Supplementary Figure 8B). The first haplotype (“101”) was almost exclusively associated with the determinate growth type I. The second haplotype (“001”) was mainly associated with growth types II and IV. The remaining three haplotypes were associated with climbing growth habit (types III and IV). An important breeding goal is to shorten DF in all growth types while maintaining other agronomic traits. Therefore, all SNPs in the region from 44.60 to 45.47 Mbp on Chr 1 were clustered, resulting in ten distinct haplotypes according to the average silhouette width (Figure 5B). As expected, the haplotypes showed strong effects on DF and explained almost 50% of the variation for DF. However, these haplotype effects were not consistent among the growth types, explaining 3.7, 36.9, 0.0, and 5.3% of the DF variation in growth types I, II, III, and IV, respectively. In addition, the effect on the remaining traits varied substantially across the haplotypes. Since only a few SNPs expressed significant pleiotropic effects, some trade-offs between traits could be removed by breaking the LD of QTL on different chromosomes (Figure 5A). In summary, the QTL on Chr 1 between 44.60 to 45.47 Mbp controlled major processes across the growth types but showed minor effects among them. Furthermore, the QTL exhibited varying pleiotropic effects on SdFe, 100SdW, and yield which can be decreased by breaking the LD between this and further QTL (e.g., Yd_7_4.86).

### 3.5. GWAS Within the Climbing Bean Germplasm

For the genetic analyses within the climbing bean germplasm, a total of 15,589 SNPs were identified in the VEC. The population structure was moderate with PC1 and PC2 explaining 19.1 and 5.8 % of the genetic variance, respectively (Supplementary Figure 9). In total, 22 significant marker-trait associations were identified in the VEC (Supplementary Table 6). Two QTL for PHI and DF were identified on Chr 5 in proximity at 38.67 and 39.34 Mbp, respectively, indicating tight linkage (Supplementary Figure 10A). Several SNPs significantly associated with SdFe and SdZn were identified on Chr 2, 4, 6, 7, and 10. Furthermore, a QTL for canning quality was identified on Chr 7 at 2.67 Mbp. No clear *p*-value inflation or deflation was observed compared to the expected *p*-value distribution (Supplementary Figure 10B). In summary, further SNPs were identified in the VEC separately to specifically improve climbing beans.

### 3.6. Genomic Prediction

The GEBV for new VEC climbing bean lines (validation set) were calculated either with parts of the VEC or with parts of all five panels as TP.

#### 3.6.1. Genomic Prediction Among Environments

**Figure 6:**
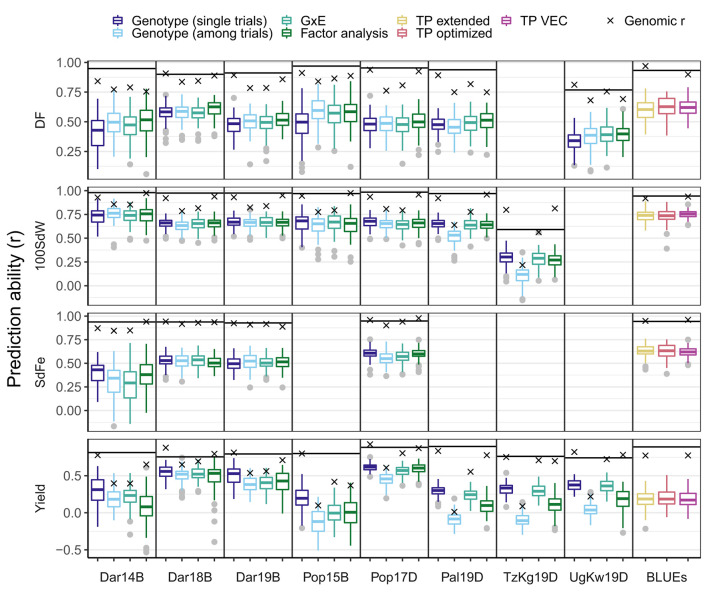
Prediction abilities for different traits and models for new lines from the climbing bean panel (VEC). The first-stage best linear unbiased estimators (BLUEs) for each trial or second-stage BLUEs across trials were used. The first-stage BLUEs models were tested accounting for either genotypic effects only, i.e., for each trial separately (single trials) or all together (among trials), genotypic x environment interaction (GxE), or correlation between trials (Factor analysis). The second-stage BLUEs were used for models which take into account genotypic effects based on the VEC (TP VEC), on all five panels (TP extended), and all five panels with optimization of the training population (TP optimized). The predicted traits were days to flowering (DF), 100 seed weight (100SdW), seed iron concentration (SdFe), and seed yield. Seed yield was scaled among panels for the models based on second-stage BLUEs to allow comparison between the different growth habits. The horizontal line is the square root of heritability indicating the heritable variance of the trait in each trial. Trials were abbreviated based on the location Darién (Dar), Palmira (Pal), Popayán (Pop) in Colombia, Kagera in Tanzania (TzKg), or Kawanda in Uganda (UgKw), the year, and the planting season (sequentially A to D). For a detailed description of each trial refer to Supplementary Table 2.

The PAs for new VEC lines within each trial and across all trials differed between the traits as well as the used model approaches (Figure 6). In general, PAs followed the heritability and the genomic *r* calculated using all available lines as TP. PAs for yield reached about *r*≈0.5 in the high-altitude locations Darién and Popayán, where climbing beans are better adapted. The PAs for yield in other locations were lower. The trials Dar14B and Pop15B showed lower PAs with higher variability because these trials comprised only 100 lines.

On average, the FA model showed the best performance for DF. The genotype model for single trials performed best for 100SdW, SdFe, and yield. The FA and GxE model reached slightly lower average PAs for SdFe and yield, respectively. The strong GxE for yield was reflected in the different marker effects among the locations (Supplementary Figure 11). The PAcc reached the highest values for 100SdW using the genotype model among trials (77.4%), DF using the FA model (67.4%), SdFe using the genotype model for single trials (63.5%), and yield using the genotype model for single trials(72.5%, Supplementary Table 7). In summary, the PAs differed among models and traits, i.e., when predicting for a single trial, the genotype model performed best, except for DF. When predicting for multiple years in one location, the FA model was promising for DF and SdFe while for yield and 100SdW, the GxE models performed best.

#### 3.6.2. Genomic Prediction Across Environments With Optimization of the Training Population

To increase PAs for new VEC lines, three different approaches were tested: when only VEC lines were in the TP (TP VEC), when all lines of the five panels were in the TP (TP extended), or when distantly related lines were excluded (TP optimized; Supplementary Figure 12). The optimized TP increased PAs for DF, SdFe, and yield (scaled among panels) when adding bush type lines of other panels and reached a PAcc of 66.8, 66.6, and 22.7% corresponding to a 0.7, 1.8, and 8.8% increase on the averaged PA, respectively (Figure 6). Regarding 100SdW, the TP optimization and extension decreased PAs slightly compared to the TP with only VEC lines. In summary, in complex traits such as SdFe and yield, the PA can be improved by adding related lines from other panels which are not in the TP even though they were tested in different trials.

## 4. Discussion

Based on the largest assembly of phenotypic and genotypic common bean data, we showed increased PAs for important traits of climbing bean by the addition of related bush type beans from other trials to the TP. In addition, the extended pool of lines, including 1,869 genotypes from distinct breeding panels, was useful to predict growth type and to increase power in the detection of QTL using GWAS (Spindel and McCouch, 2016). Hence, this comprehensive study provides a solid basis to harness the large genetic diversity of common bean germplasm and to implement marker-assisted and genomic selection strategies for more efficient climbing bean breeding.

### 4.1. QTL Across Breeding Panels

For all studied traits, QTL with clear effects on the phenotypes in all five breeding panels were detected (Figure 4). This diverse joint group of panels with fast LD decay enabled the identification of SNPs tightly linked to the causal loci while controlling for population structure (Sul et al., 2018; Huang et al., 2019). On the one hand, several QTL for growth habit and DF were confirmed from previous studies. On the other hand, especially for SdFe and 100SdW, new QTL and candidate genes were identified.

#### 4.1.1. Previously Described and New QTL for DF and Growth Habit

Considering significant marker-trait associations less than 1 Mbp away from previously reported positions, QTL for DF and growth habit were confirmed on Chr 1, 4, 9, and 11. In addition, on Chr 6, 7, and 8, significantly associated SNPs were mapped to a distance of 1 to 4 Mbp from previously reported QTL. The terminal flowering gene *PvTFL1y* (*fin* locus identified as Phvul.001G189200 on Chr 1 at 44.85 Mbp) (Norton, 1915; Koinange et al., 1996; Kwak et al., 2008; Repinski et al., 2012; González et al., 2016) was confirmed by DF_1_44.60 (Table 1). The *phytochrome A* gene (*Ppd* locus identified as Phvul.001G221100 on Chr 1 at 47.64 Mbp) conferring photoperiod sensitivity (Coyne and Schuster, 1974; Gu et al., 1998; Kamfwa et al., 2015; Weller et al., 2019) was tightly linked to GH_1_47.43. In agreement, the haplotype “101” constructed in the region from 43.71 to 45.37 Mbp almost exclusively differentiated between determinate and indeterminate growth types (Supplementary Figure 8B). Thus, multiple SNPs are required to determine growth type including the allelic version of the *PvTFL1y* gene.

In the region of the two identified SNPs for growth habit on Chr 4 (GH_4_1.42 and GH_4_1.92), a QTL associated with climbing ability and plant height was previously reported (linked to Pvctt001 marker at 0.51 Mbp) (Checa and Blair, 2008). Furthermore, in proximity to GH_6_29.43, a QTL for DF was previously reported at 31.6 Mbp (Raggi et al., 2019). The terminal flowering gene *PvTFL1z* (Phvul.007G229300 a homolog of *PvTFL1y*) was located on Chr 7 at 35.31 Mbp (Kwak et al., 2008). In our study, GH_7_38.99 was detected proximal to PvTFL1z. On the upper arm of Chr 8 at 4.9 Mbp, another QTL for DF was reported previously (Raggi et al., 2019). Similarly, we detected GH_8_2.11 at less than 3 Mbp distance. A second fin locus (*fin'*) on Chr 9 was tagged by molecular markers at 13.39 Mbp (de Campos et al., 2011) and at around 20 cM (González et al., 2016). This *fin'* locus is probably tagged by GH_9_13.92. A QTL for DF on Chr 11 at around 9 cM reported in Bhakta et al. (2017) was linked to GH_11_1.01 (at around 8.8 cM). A new QTL for growth habit, GH_2_40.05 was identified with one SNP variant exclusively associated to climbing beans, however, with a low MAF of 5%. Newly identified SNPs for growth habit with lower LOD scores have to be interpreted carefully due to the observed *p*-value inflation, indicating remaining population structure. In conclusion, the joint analysis consisting of diverse common bean populations showed high detection power for DF and growth habit QTL. Four to potentially seven QTL known from previous studies and several new QTL for DF and growth habit were identified and tagged by tightly linked SNP markers.

#### 4.1.2. QTL for SdFe and Their Independence From Growth Habit

Four meta-QTL were previously reported for SdFe on Chr 1 (between 43.3 and 48.5 Mbp), on Chr 6 (between 28.2 and 29.5 Mbp), on Chr 9 (between 11.7 and 13.5 Mbp), and Chr 11 (between 2.3 and 5.3 Mbp) (Izquierdo et al., 2018). All four QTL fall right onto or next to QTL for growth habit identified in the current study. Since climbing beans in general exhibit a higher SdFe (Blair et al., 2010; Petry et al., 2015), the previously reported QTL is probably confounded with population structure. The effects of these QTL on SdFe were also detected in our analysis but the associations did not exceed the 5% significance threshold (Supplementary Figure 7B). A QTL for SdZn was recently reported on Chr 1 at 49.37 Mbp in European landraces next to *PvTFL1y*, suggesting a genetic linkage between DF and SdZn (Caproni et al., 2020). From the three major QTL for SdFe detected in our study, two were identified for the first time and SdFe_6_22.37 was previously reported in proximity at 22.8 Mbp (Diaz et al., 2020). The identified SNPs for SdFe on Chr 2 and 6 showed an effect in all three evaluated breeding panels and are thus independent of the growth habit (Figure 4). The QTL at the end of Chr 2 (SdFe_2_46.07) is of particular interest because it was linked to a QTL for DPM in this study and a pleiotropic QTL affecting DF, DPM, 100SdW, and yield in the VEF (Keller et al., 2020). In conclusion, various QTL for SdFe reported previously seemed to be confounded with growth habit, while our joint analysis allowed us to detect new SNPs associated with SdFe across different breeding panels.

#### 4.1.3. Candidate Gene Identification for SdFe and 100SdW

Four new candidate genes were identified for SdFe and 100SdW: on Chr 6 at 22.18 Mbp, in a distance of less than 200,000 bp from the SNP most significantly associated to SdFe (SdFe_6_22.37), the Phvul.006G113100 gene was annotated as a homologous to a ferric-chelate reductase, reported to be involved in iron uptake from the soil (Robinson et al., 1999; Wu et al., 2005; Jeong et al., 2008; Asard et al., 2013). In proximity, less than 125,000 bp away from SdFe_2_46.07 and SdFe_9_36.80, the genes Phvul.002G292900 and Phvul.009G247600, respectively, were annotated. These two genes putatively express *Atox1*-related copper transport proteins, which are involved in copper and iron homeostasis (Himelblau et al., 1998; Puig et al., 2007). A QTL for copper and iron uptake was shown previously to have close genetic linkage (Waters and Grusak, 2008). Regarding 100SdW, a putative asparagine synthetase (Phvul.006G188400) on Chr 6 at 28.87 Mbp, less than 30,000 bp away from SdW_6_28.90, showed major effects in all breeding panels (Figure 4). The asparagine synthetase remobilizes nitrogen from sources to sinks and was reported to increase seed weight and soluble protein content in Arabidopsis seed (Gaufichon et al., 2016, 2017). In conclusion, for the major QTL for SdFe and 100SdW, plausible candidate genes were identified whose putative functions remain to be further validated.

### 4.2. QTL Detected Within Breeding Panels

Several QTL were identified only in the VEC, e.g., a pleiotropic QTL for DF and PHI on Chr 5 between 38.68 and 39.34 Mbp. The QTL for PHI differed from QTL previously identified in bi-parental bush type populations (Mukeshimana et al., 2014; Diaz et al., 2018). The PHI was weakly and positively correlated to DF and yield (Supplementary Figure 3). This pleiotropic effect of the identified QTL might be related to the time from flowering until harvest which could affect DF and PHI. Regarding DF, the major QTL tagged by DF_1_44.60 was mapped more closely to *PvTFL1y* in the current joint analysis than in a separate analysis of the ADP and VEF at 48.34 and 49.72 Mbp, respectively (Kamfwa et al., 2015; Keller et al., 2020). In agreement, the LD decay of the combined panel was faster than that of the separate panels. Furthermore, we concluded that the genetic control of flowering is different on the single SNP level (Figure 4) and haplotype level among the growth types (Figure 5B). The major QTL for DF on Chr 1 showed no effect within growth type III lines. One possible explanation is that DF within the climbing growth habit is regulated by additional genes or growth habit specific alleles. Additionally, haplotype 1 showed increased yield for growth types I and II while the effect on DF differed. It demonstrates that DF and yield are loosely linked as suggested earlier by White et al. (1992). Similarly, the QTL for SdFe on Chr 2 at 2.91 and 48.86 Mbp were valid only for the VEC and were not detected in the joint analysis suggesting some specific alleles were present only in the climbing bean panel.

### 4.3. Genomic Predictions Across and Within Breeding Panels

Using only VEC lines as TP in the different modeling approaches, the FA model performed well for DF and SdFe, showing the importance of covariance between trials for those traits (Figure 6). Such FA models work well for different environments, where some lines were already tested, were previously reported for sweet cherry (*Prunus avium* L.) (Hardner et al., 2019). The PAcc for yield of the genotype model using the first- and second-stage BLUEs among all environments were lower in comparison to the FA model and GxE model which took environmental differences into account. In agreement, the high GxE was visible in the correlations between the trials which reached values from –0.41 to 0.64 (Figure 1B). Those interactions were additionally reflected in the relatively high PA of the GxE model in yield and in the differing marker effects among locations (Figure 6 and Supplementary Figure 11). Comparing different traits, the correlations between yield and DPM in bush type beans altered when changing from low- to high-altitude locations (Diaz et al., 2018). Therefore, the single-trial genotype model and the GxE model, which account for location effects such as high- and lowland, worked best for the predictions of yield. Similarly, high GxE for yield was shown before in the VEF under different environmental conditions (Keller et al., 2020). Across all trials, the TP optimization turned out to be a promising strategy to predict the performance of new climbing bean lines. For complex traits such as SdFe and yield, optimizing the TP with related lines from different panels tested under different conditions improved the PA by 1.8 and 8.8%, respectively. Especially for smaller breeding programs or new breeding panels, it is, therefore, advisable to optimize the TP even when the added lines were tested in different field trials.

## 5. Conclusion

The benefits of introducing genes conferring climbing ability into new breeding lines are limited because growth habits are fixed in specific production systems and their modification would have pleiotropic effects, most likely affecting traits like DF and SdFe negatively. The most significant QTL for growth habit at the end of Chr 1 was pleiotropic. However, this QTL was in LD with several other QTL which can be selected separately since they were located on different chromosomes (Figure 5A). In addition, this QTL had no effect on common beans of the growth type III. Regarding other QTL, we detected stable SdFe and yield QTL which showed effects in all tested panels without significant pleiotropic effects. The identified markers were validated in very diverse germplasm including all growth types and both gene pools. The resulting fast LD decay allowed mapping of QTL more precisely than was achieved in separated panels. Genomic prediction models were established across populations enabling the selection and hybridization of the best lines of all populations to combine favorable alleles. Especially for yield, GxE needs to be modeled when breeding for different environments. The large common bean diversity presented in this study was used to identify markers across populations and to establish and improve prediction models. The joint population will provide a basis to exploit this genetic diversity and will contribute to the quick and targeted development of new (climbing bean) lines.

## Data Availability Statement

The original contributions presented in the study are publicly available. This data can be found here: https://doi.org/10.7910/DVN/RLAWYN.

## Author Contributions

BR and BS conceived the study. CM, AP-B, HB, WA, SM, JM, TP, JB, and PM conducted the experiments and provided the phenotypic data. BK, DA-S, and JA performed modeling, data analysis, and interpretation. DA-S prepared the genotypic data. BK drafted the manuscript, which was improved by DA-S, BS, PM, TP, and BR. All authors approved to the manuscript submission.

## Funding

The authors would like to thank the COOP Research Program on Sustainability in Food Value Chains of the ETH Zurich World Food System Center and the ETH Zurich Foundation for supporting this project. The COOP Research Program was supported by the COOP Sustainability Fund. The work was additionally founded by Tropical Legumes III-Improving Livelihoods for Smallholder Farmers: Enhanced Grain Legume Productivity and Production in Sub-Saharan Africa and South Asia (OPP1114827), and by the AVISA-Accelerated varietal improvement and seed delivery of legumes and cereals in Africa (OPP1198373) projects funded by the Bill & Melinda Gates Foundation. The Norman Borlaug Cooperative Research Initiative (NBCRI) Grain Legumes Project of the US Agency for International Development, Project No. 0210-22310-004-96R supported the Tanzanian trials in Arusha and Mbeya. We would like to thank USAID for their contributions through the CGIAR Research Program on Grain Legumes and Dryland Cereals. Open access publication is kindly covered by ETH Zurich.

## Conflict of Interest

The authors declare that the research was conducted in the absence of any commercial or financial relationships that could be construed as a potential conflict of interest.

## Publisher's Note

All claims expressed in this article are solely those of the authors and do not necessarily represent those of their affiliated organizations, or those of the publisher, the editors and the reviewers. Any product that may be evaluated in this article, or claim that may be made by its manufacturer, is not guaranteed or endorsed by the publisher.
