# Supplementary material for: Improving Association Studies and Genomic Predictions for Climbing Beans With Data From Bush Bean Populations
Source: Front Plant Sci. 2022 Apr 25;13:830896. doi: 10.3389/fpls.2022.830896 (PMC9085748; doi:10.3389/fpls.2022.830896)
Supplement: Supplementary file 1 [file Data_Sheet_1.pdf]

Supplementary material

1 SUPPLEMENTARY DATA

All raw data for this study is available under: `https://doi.org/10.7910/DVN/RLAWYN`.

2 SUPPLEMENTARY FIGURES

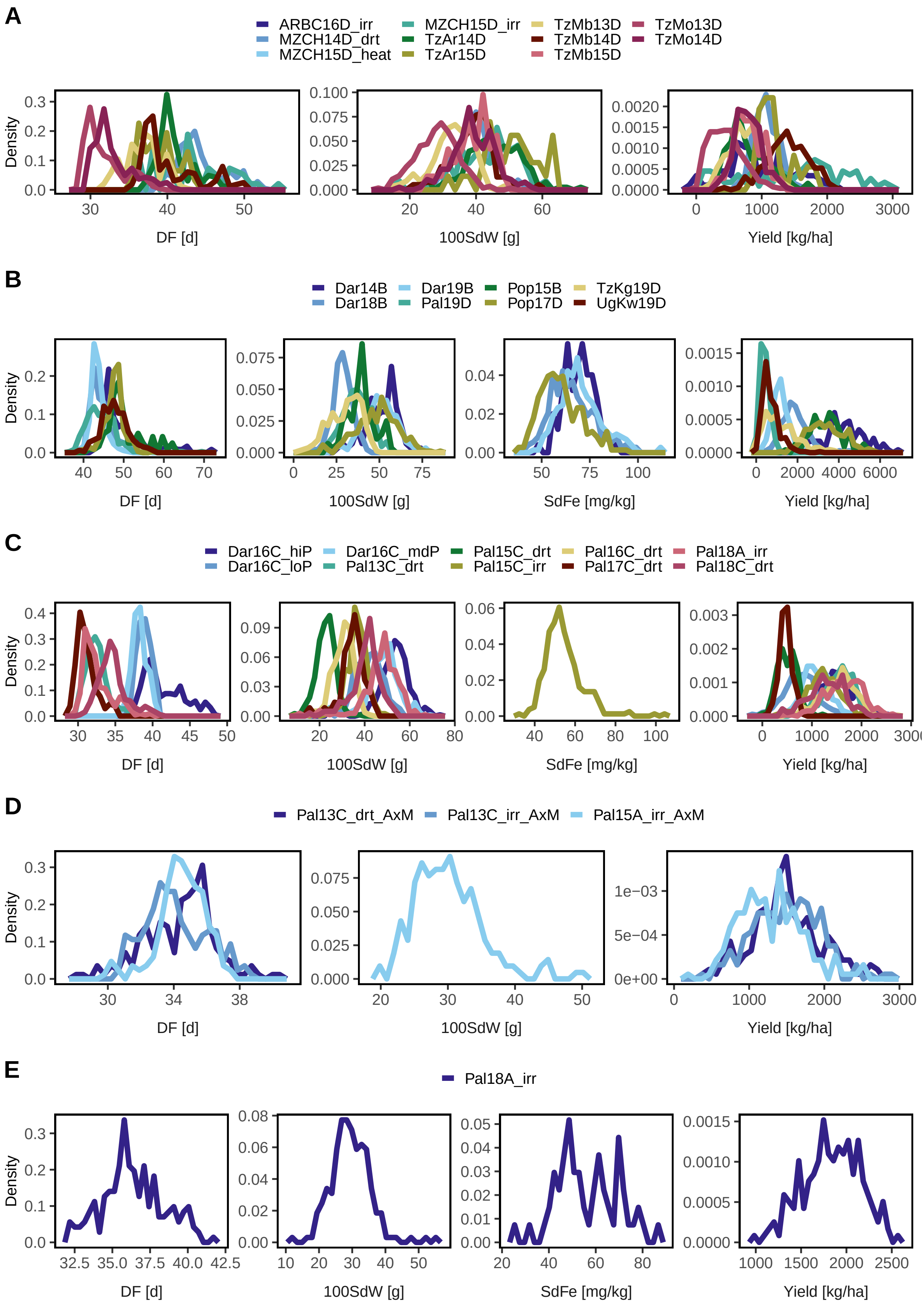

Supplementary Figure 1. Density plots of phenotypic data for five bean breeding panels. The panels, ADP (A), VEC (B), VEF (C), AxM (D), and MIP (E) were evaluated for days to flowering (DF), 100 seed weight (100SdW), seed iron concentration (SdFe) and seed yield. For the description of each trial see Supplementary Table 2, Supplementary Table 3 and Supplementary Table 4.

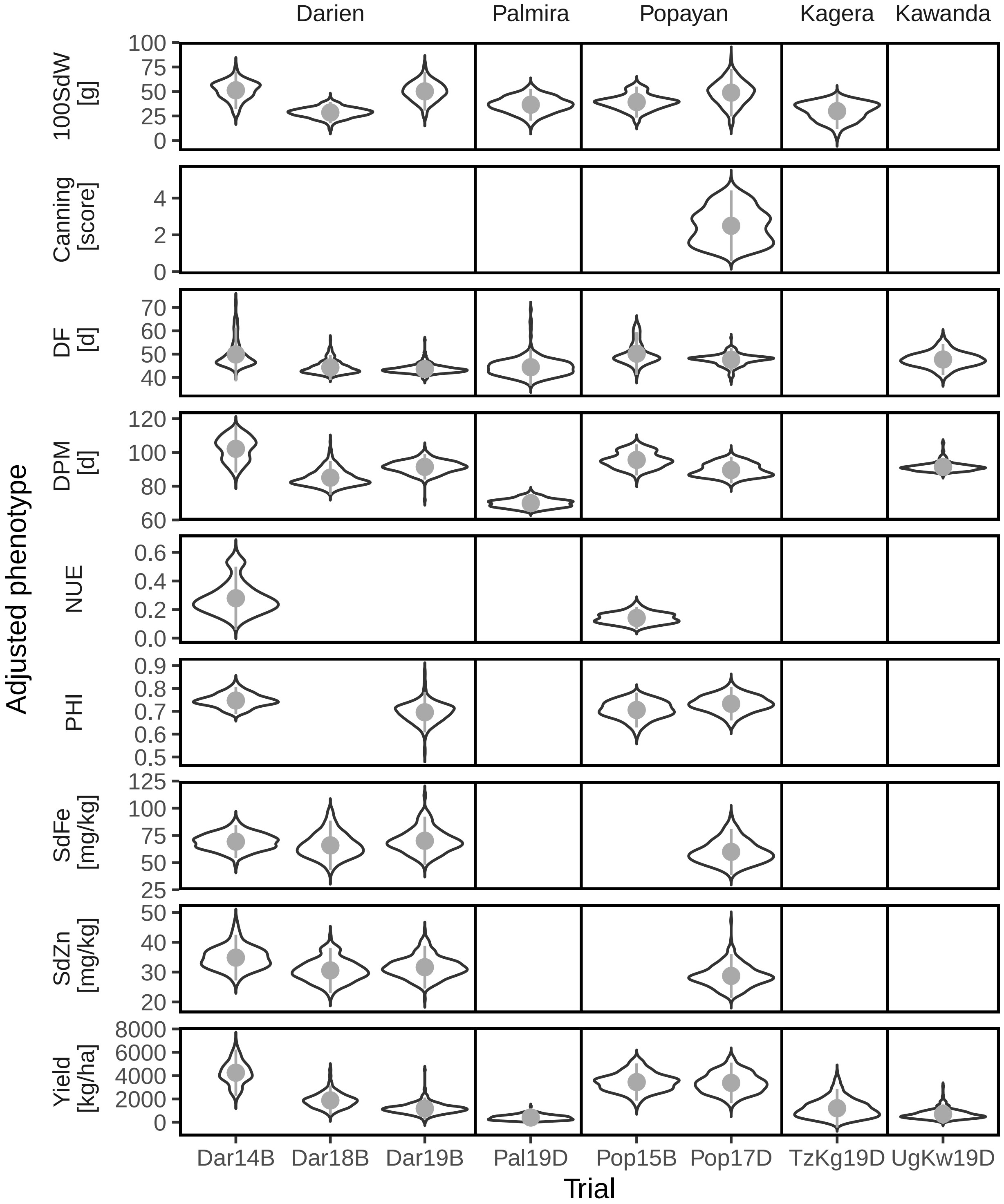

Supplementary Figure 2. Phenotypes of the climbing bean panel (VEC) evaluated in eight field trials at five locations. The 290 lines were evaluated for 100 seed weight (100SdW), canning quality, days to flowering (DF), days to physiological maturity (DPM), nitrogen use efficiency (NUE), pod harvest index (PHI), seed iron concentration (SdFe), seed iron zinc (SdZn) and seed yield. The trials in 2014 and 2015 (Dar14B and Pop15B) were evaluated by Barbosa et al. (2018). Trials were abbreviated based on the location Darién (Dar), Palmira (Pal), Popayán (Pop) in Colombia, Kagera in Tanzania (TzKg), or Kawanda in Uganda (UgKw), the year and the planting season (sequentially A to D). For a detailed description of each trial see Supplementary Table 2.

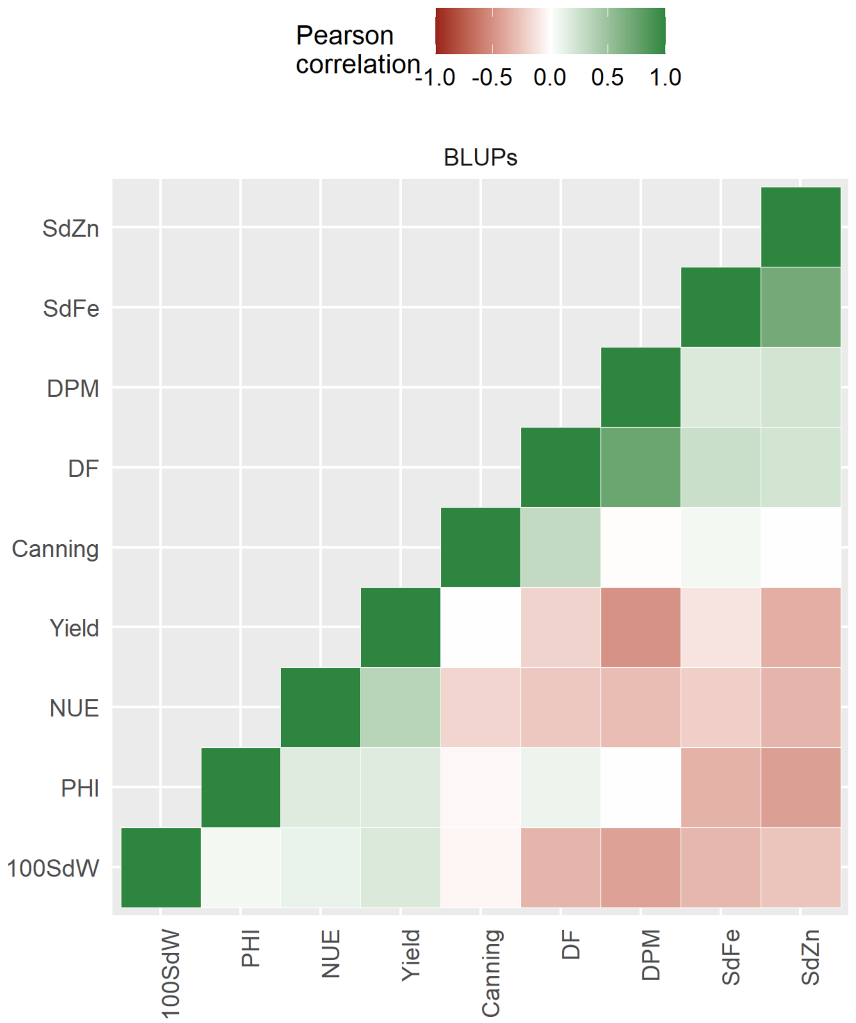

Supplementary Figure 3. Correlations of second-stage BLUPs in the climbing bean panel (VEC) for each trait. The 290 lines were evaluated for 100 seed weight (100SdW), canning quality, days to flowering (DF), days to physiological maturity (DPM), nitrogen use efficiency (NUE), pod harvest index (PHI), seed iron concentration (SdFe), seed iron zinc (SdZn) and seed yield. The NUE data was taken from Barbosa et al. (2018).

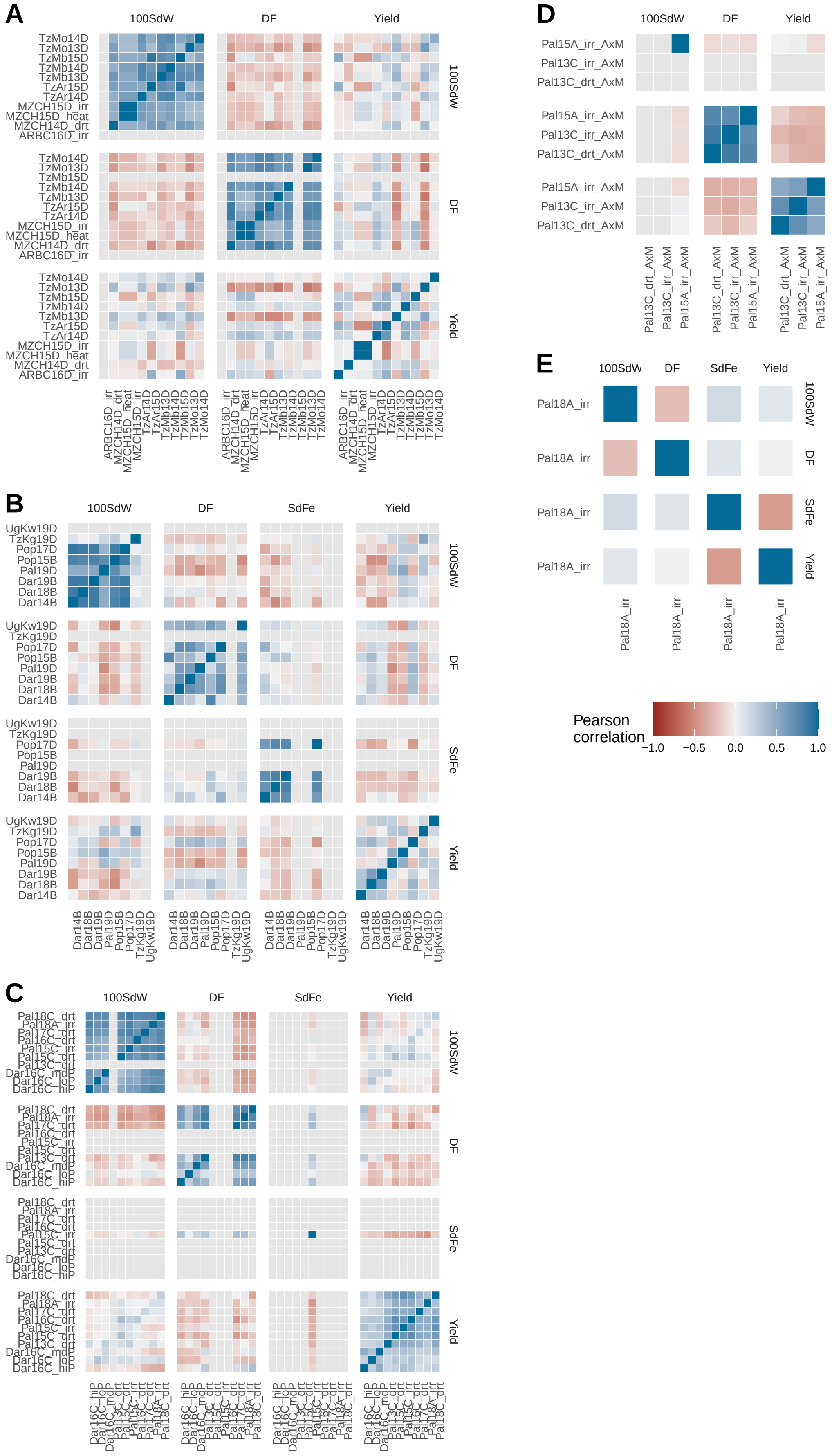

Supplementary Figure 4. Pearson correlation coefficients of phenotypes between trials and traits for five bean breeding panels. The panels, ADP (A), VEC (B), VEF (C), AxM (D), and MIP (E), were evaluated for days to flowering (DF), 100 seed weight (100SdW), seed iron concentration (SdFe) and seed yield. For the description of each trials see Supplementary Table 2, Supplementary Table 3 and Supplementary Table 4.

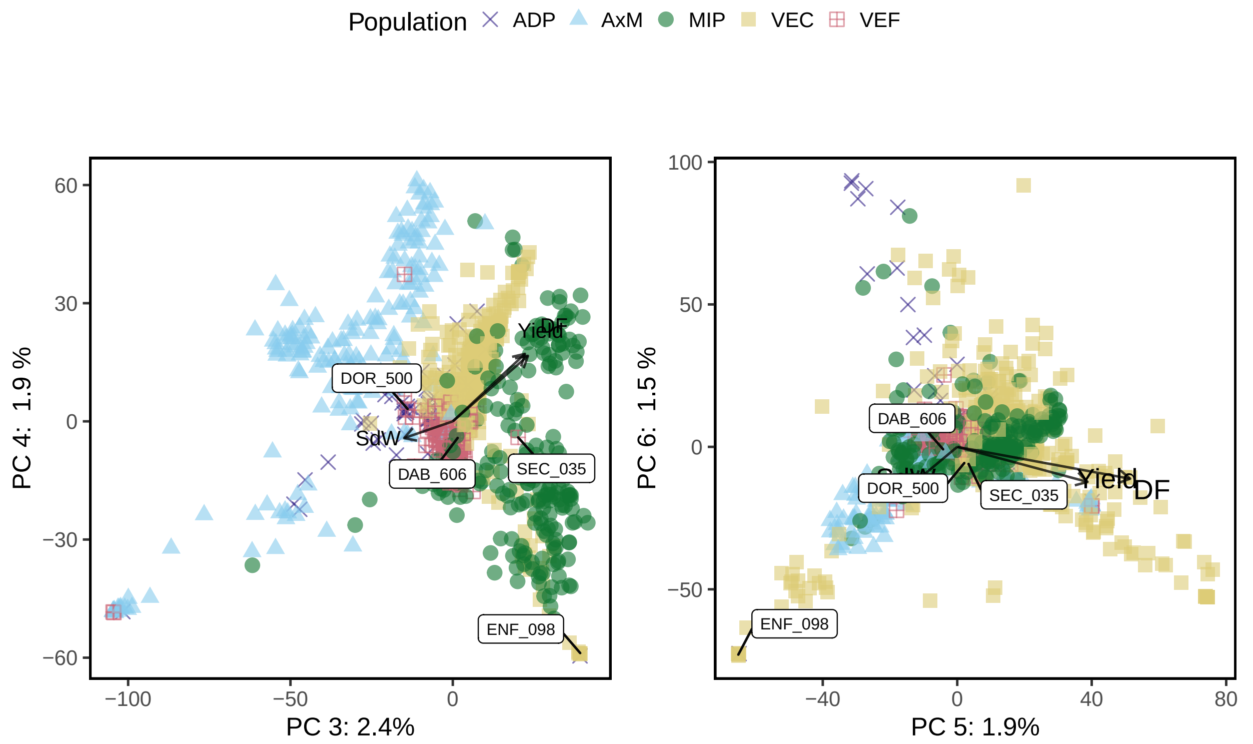

Supplementary Figure 5. Principal component (PC) 3 to 6 for the five bean breeding panels based on 14,913 SNP markers. The arrows show days to flowering (DF), 100 seed weight (100SdW) and seed yield as supplementary traits. Their cosines indicate the correlation with PC axes and their length approximate the standard deviation of the variable. The extreme lines from the PC 1 axis are labeled.

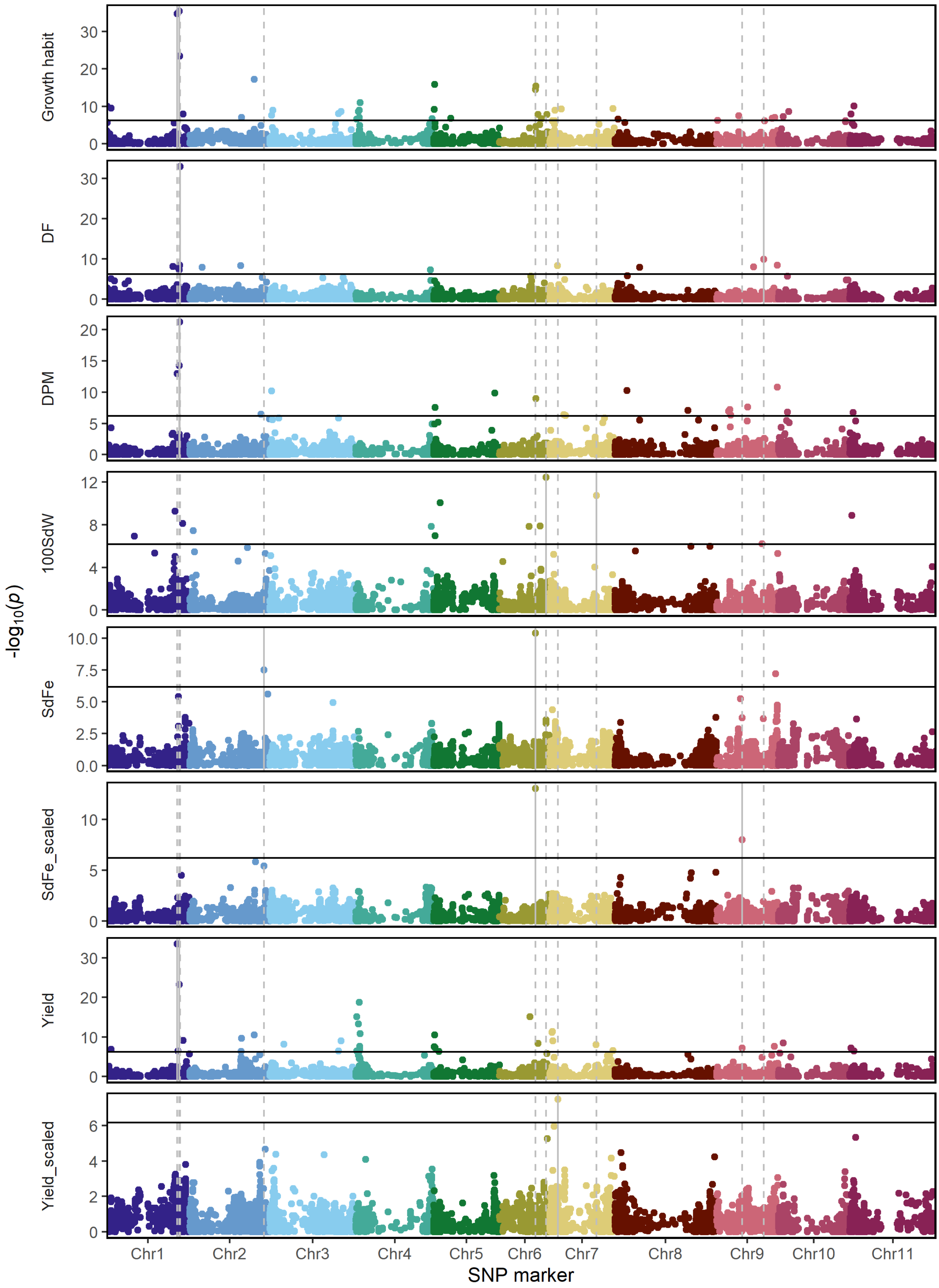

Supplementary Figure 6. Manhattan plots for all traits as revealed by to genome-wide association studies using best linear unbiased estimators across all five breeding panels. The traits growth habit, days to flowering (DF), days to physiological maturity (DPM), 100 seed weight (100SdW), seed iron concentration (SdFe), seed iron zinc (SdZn) and seed yield were evaluated. Seed yield and SdFe were additionally scaled among panels.

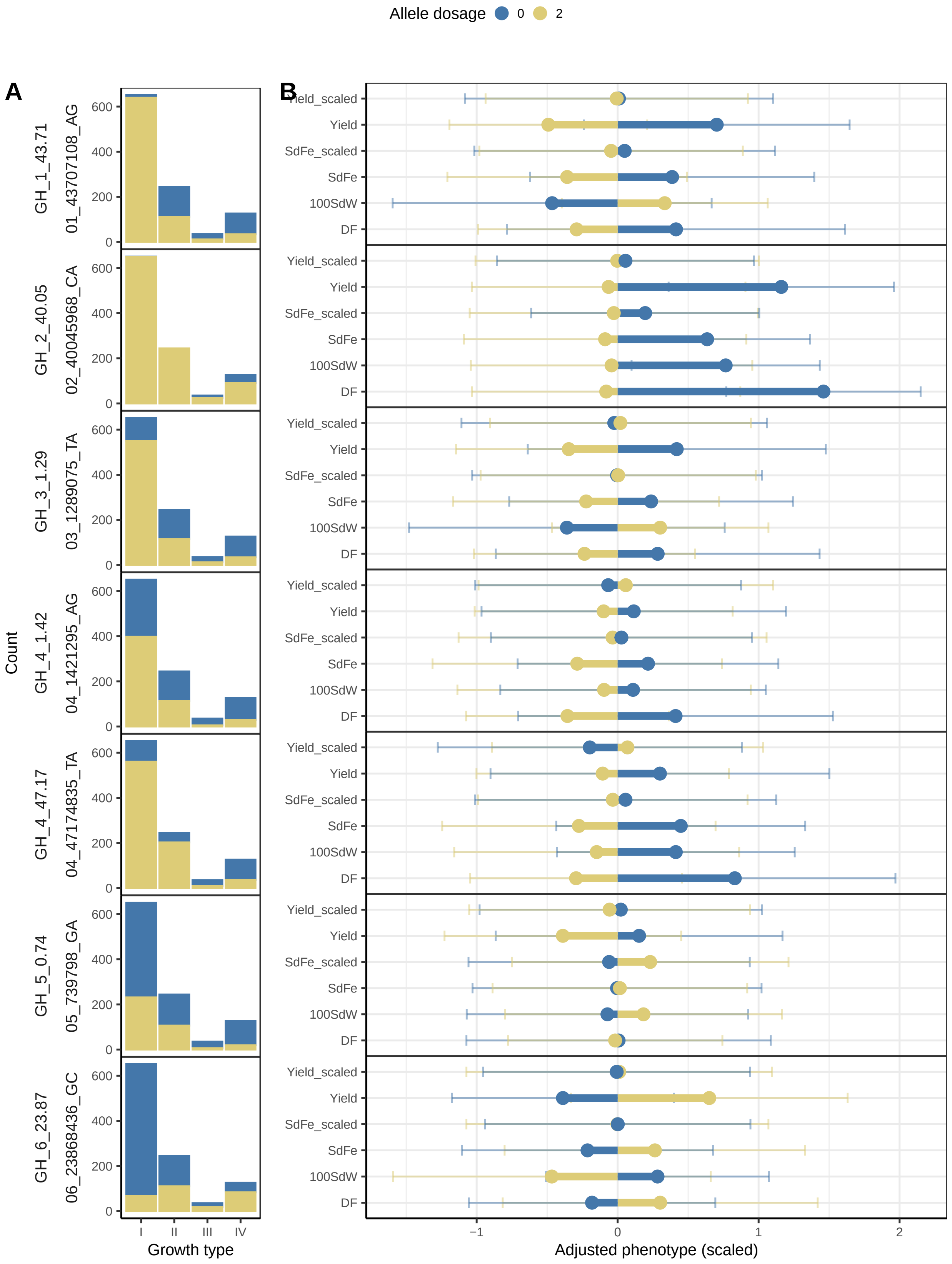

Supplementary Figure 7. The effect of allele dosage (0 or 2 alternative alleles) on the phenotypes related to growth types from selected marker-trait associations. A: The significant SNPs for growth habit divide growth types I (determinate bush type), type II (indeterminate bush), type III (determinate climber) and type IV (indeterminate climber) in different proportions. B: The SNPs showed pleiotropic effects on days to flowering (DF), 100 seed weight (100SdW), seed iron concentration (SdFe) and seed yield. Seed yield and SdFe were additionally scaled among the panels. The pleiotropic SNP effects were scaled for each trait in order to make them comparable.

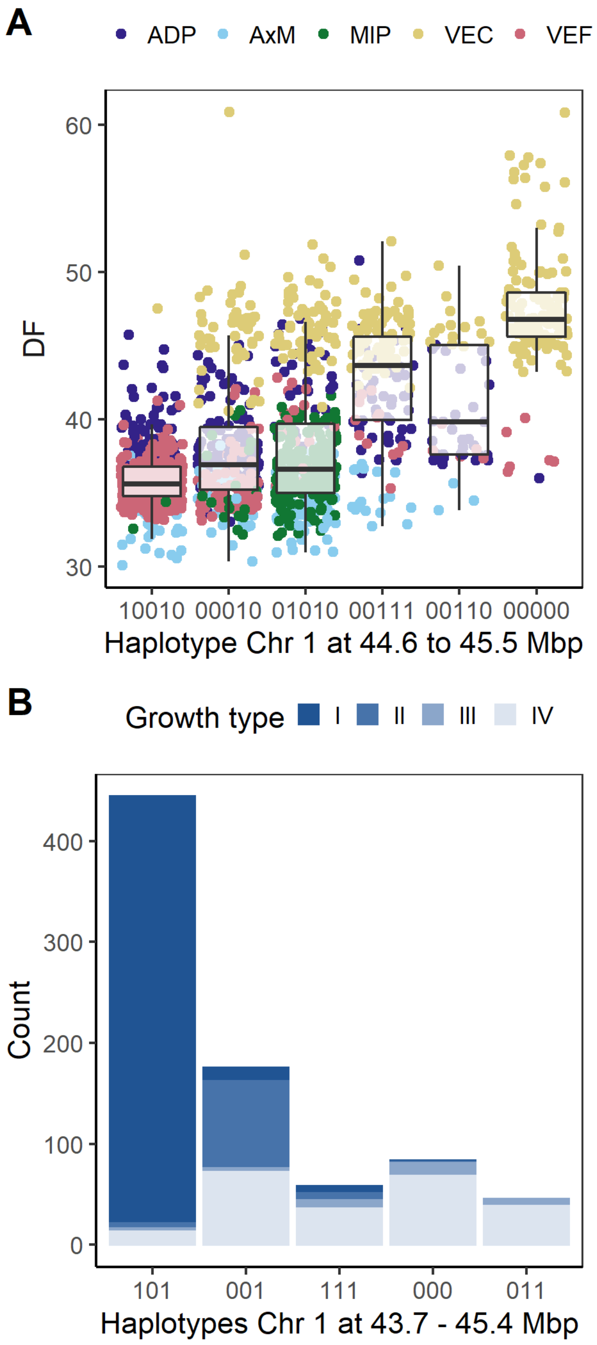

Supplementary Figure 8. Effect of haplotypes constructed with SNPs significantly associated with days to flowering (DF) and growth habit at the end of chromosome (Chr) 1. A: The boxplots show the haplotype effects of SNPs significantly associated with DF. B: The haplotypes of SNPs significantly associated with growth habit divide growth types I (determinate bush type), type II (indeterminate bush), type III (determinate climber) and type IV (indeterminate climber) in different proportions.

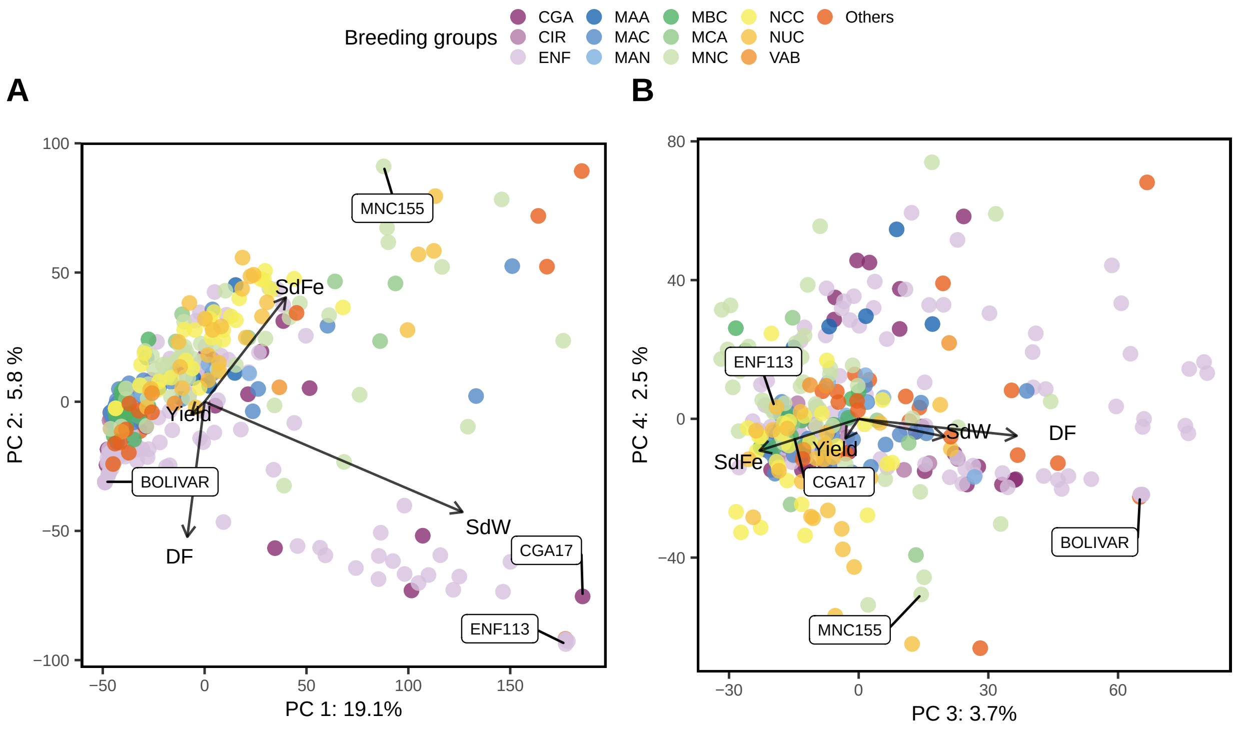

Supplementary Figure 9. Principal component (PC) analysis for the climbing bean panel (VEC) based on 15,589 SNP markers. A: The PC 1 to 2 and B: the PC 3 to 4 visualize the population structure of the VEC lines belonging to different breeding groups. The arrows show days to flowering (DF), 100 seed weight (100SdW) and seed yield as supplementary traits. Their cosines indicate the correlation with PC axes and their length approximate the standard deviation of the variable. The lines with extreme values on the PC 1 and PC 2 axis are labeled. For a description of the different breeding groups see Supplementary Table 1).

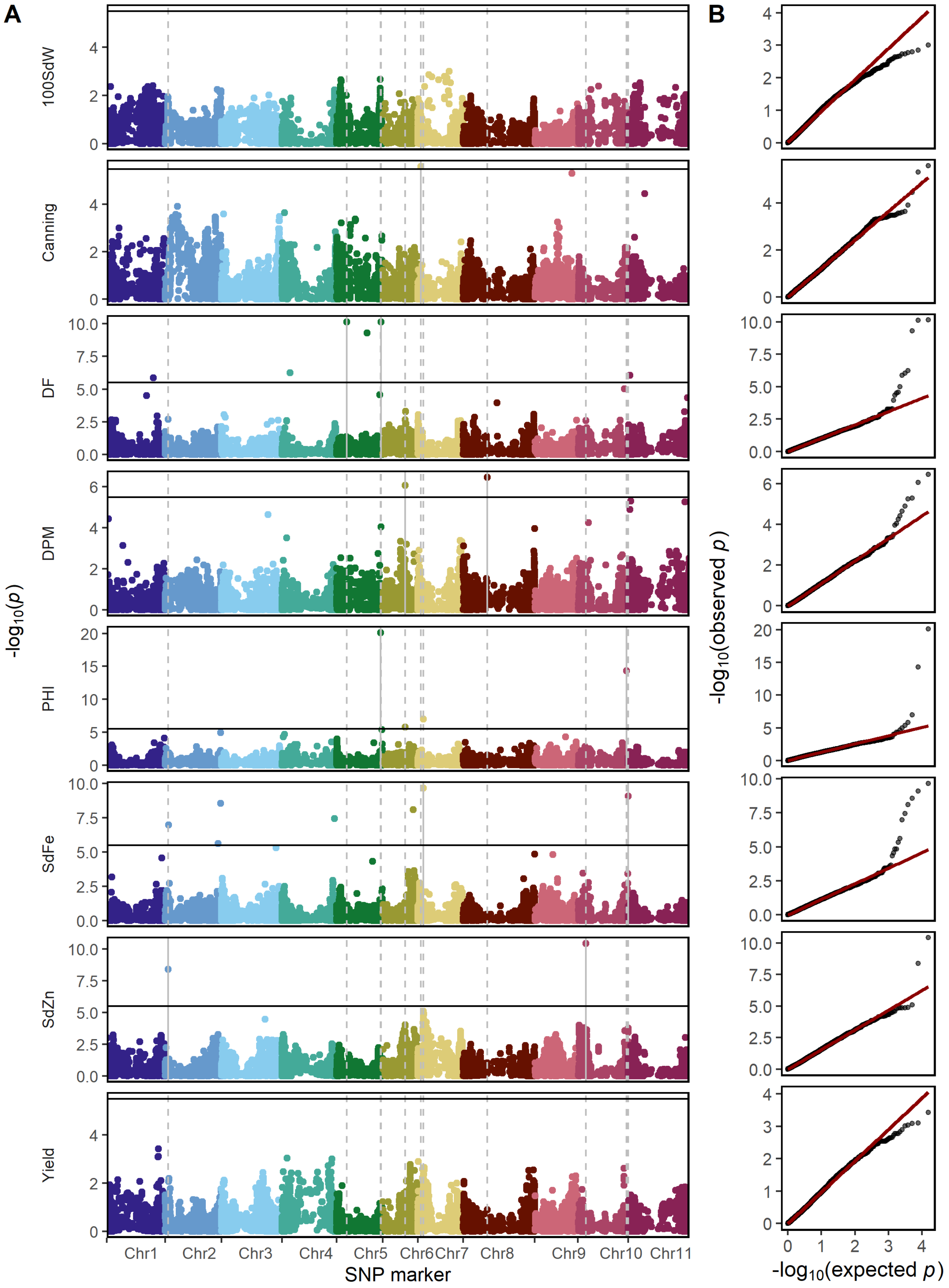

Supplementary Figure 10. Genome-wide association studies carried out for the the climbing bean panel (VEC) in order to identify associated genetic loci. A: Manhattan plots show the genetic associations with 100 seed weight (100SdW), canning quality, days to flowering (DF), days to physiological maturity (DPM), pod harvest index (PHI), seed iron concentration (SdFe), seed iron zinc (SdZn) and seed yield. The horizontal black lines show the Bonferroni corrected significance threshold at the 5% level. B: Quantile distribution plots show the deviation of expected to observed p values of SNP to trait associations for each trait.

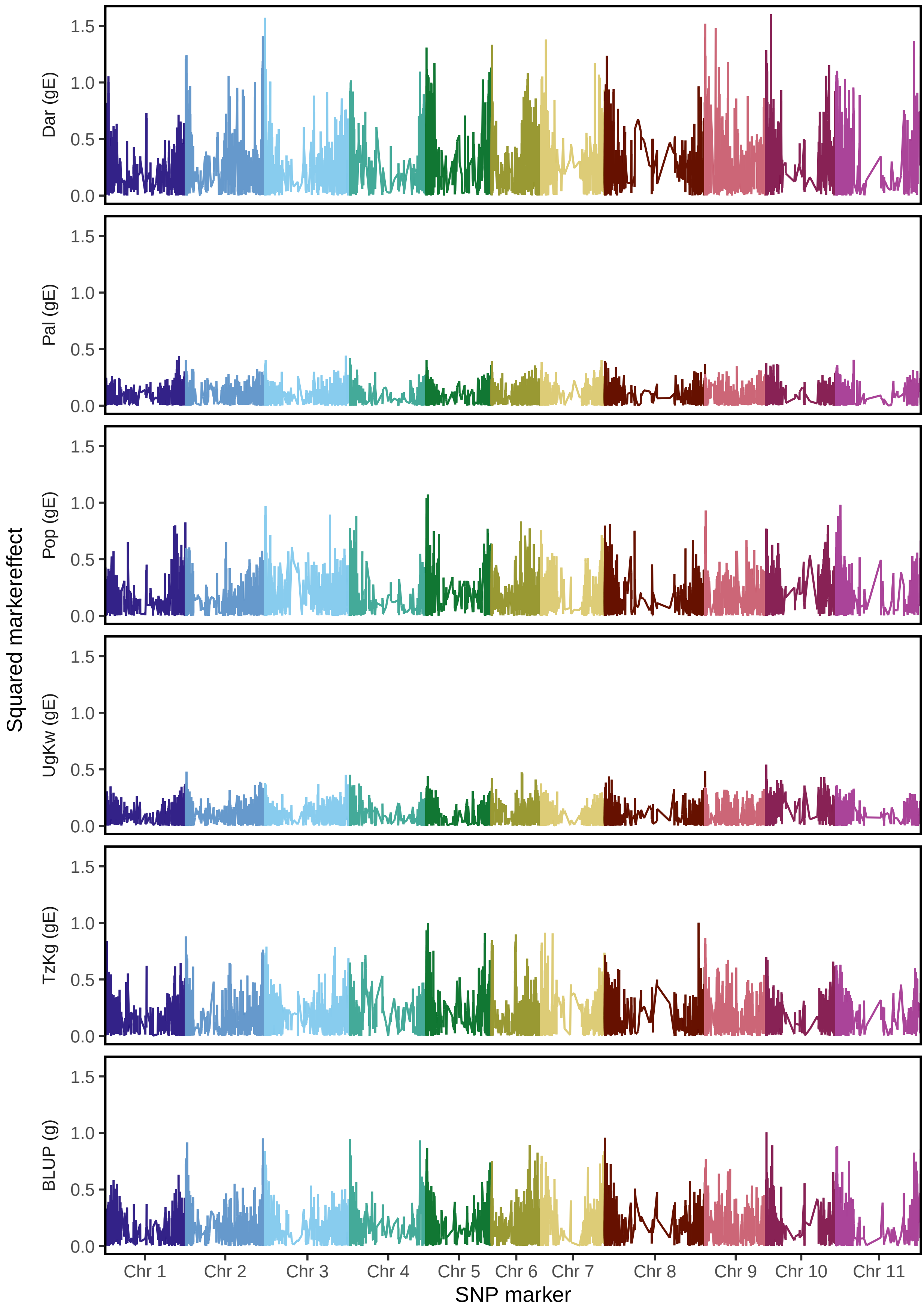

Supplementary Figure 11. Marker effects for yield among the different locations are shown for the climbing bean panel (VEC). Marker effects were calculated for the genotypic effects (BLUPs) and genotype by environment interactions (gE) for each location according to model (3) using all available lines in the training population.

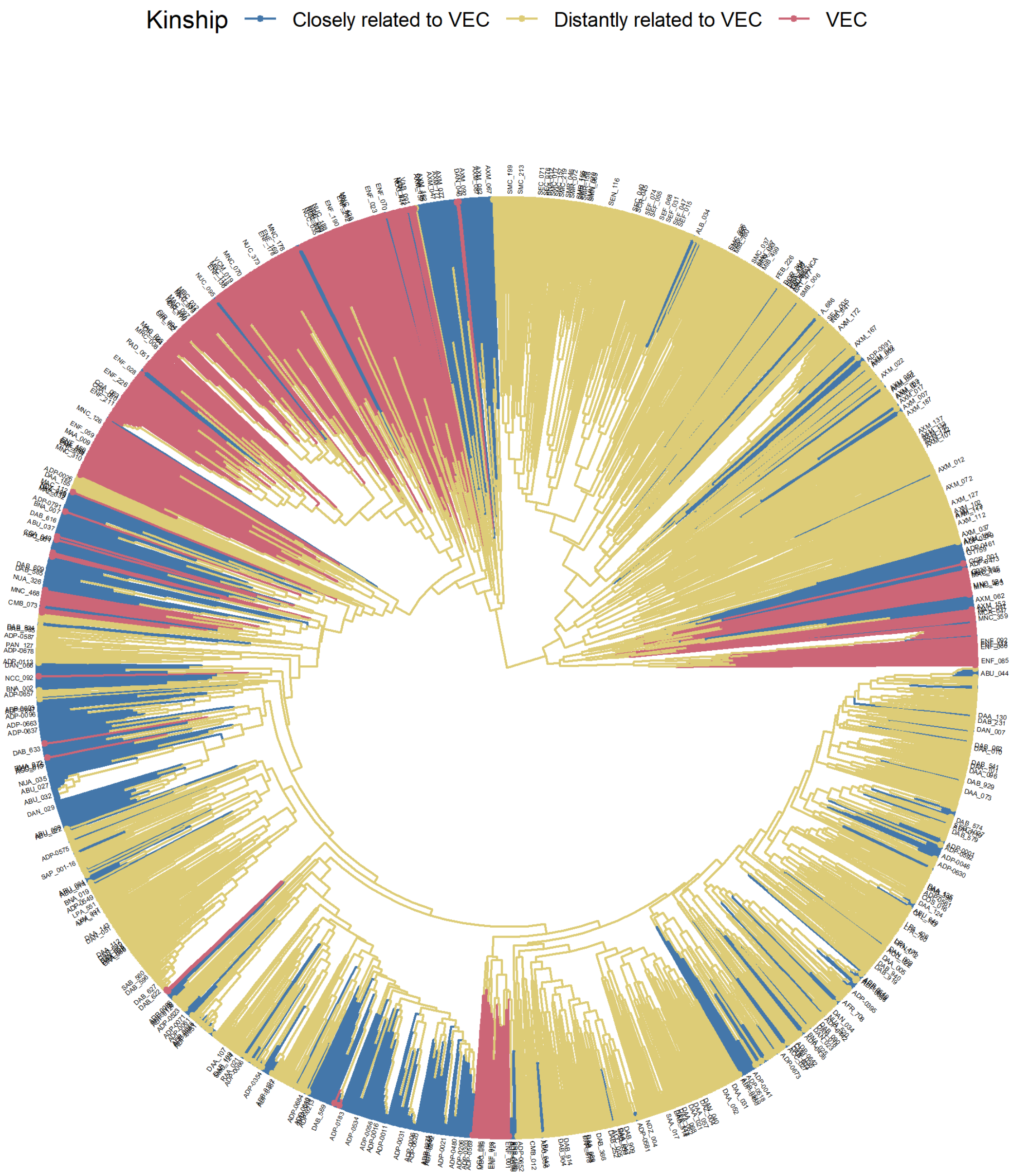

Supplementary Figure 12. The climbing bean panel (VEC) and its closest relatives among four other breeding panels shown by a dendrogram. The degree of kinship was used to optimize the training population for genomic prediction in a total of 1,869 lines.

3 SUPPLEMENTARY TABLES

Supplementary Table 1. Composition of the climbing bean panel (VEC) is shown with breeding group, cultivar or accession, their names and the number of lines (No.).

Group	Name	No.
Accessions	G14519 (from the US), G13595 (M-112, Cuba), G19833 (Peru), G2333	4
CGA	Climbers Good Agronomic	19
CIR	Climbers Interspecific Root rots	21
Cultivars	AHSABANERO, B ROJA, BOLIVAR, C MOSQUENO, C ROJO, D MORENO	6
ENF	*Enfermedades* in Spanish for disease	58
MAA	Marker-Assisted Anthracnose resistance	5
MAC	Mid-Altitude Climbing beans	31
MAN	Mid-altitude Andean Nutrition climbing beans	7
MBC	Mid-altitude Bean common mosaic necrosis virus resistant Climbing beans	14
MCA	Mid-altitude Climbing beans with Angular leaf spot resistance	7
MNC	Mid-altitude Nutritionally enhanced Climbing beans	49
NCC	Nutrition Common Climbing beans	27
NUC	Nutritionally enhanced large seeded Climbing beans	26
Others	Other breeding groups	14
VRA	Red-climbing bean for highlands	2

Supplementary Table 2. Description of the field sites and trials carried out for the climbing bean panel (VEC). Trials were abbreviated based on the location Darién (Dar), Palmira (Pal), Popayán (Pop) in Colombia, Kagera in Tanzania (TzKg), or Kawanda in Uganda (UgKw), the year and the planting season (sequentially A to D). Harvested plot size can differ from the indicated planted plot size. The data for Dar14B and Pop15B was available from Barbosa et al. (2018). All trails were rainfed.

Trial	Year	Location	GPS location	Elevation (masl)	Soil type	Plot length (m)	Plot width (m)	Plot rows (No.)	Replicate (No.)	Conditions	Lines (No.)
Dar14B	2014	Darién, Colombia	3°53’31”N 76°31’00”W	1491	Inceptisol	2.5	0.95	1	3	Rainfed	98
Dar18B	2018	Darién, Colombia	3°53’31”N 76°31’00”W	1491	Inceptisol	2.5	0.95	1	3	Rainfed	290
Dar19B	2019	Darién, Colombia	3°53’31”N 76°31’00”W	1491	Inceptisol	2.5	0.95	1	3	Rainfed	290
Pop15B	2015	Popayán, Colombia	2°25’39”N 76°37’17”W	1750	Inceptisol	2.0	0.95	1	3	Rainfed	98
Pop17D	2017	Popayán, Colombia	2°25’39”N 76°37’17”W	1750	Inceptisol	2.0	0.95	1	3	Rainfed	290
Pal19D	2019	Palmira, Colombia	3°30’03.0”N 76°21’03.5”W	965	Mollisol	2.2	0.95	1	3	Rainfed	290
TzKg19D	2019	Kagera, Tanzania	1°24’56.5”S 31°46’48.8”E	1320	n.a.	3.0	0.6	1	3	Rainfed	290
UgKw19D	2019	Kawanda, Uganda	0°24’49”N 32°31’59”E	1190	n.a.	3.0	0.6	1	3	Rainfed	290

Supplementary Table 3. Description of the field sites and trials carried out for the Andean diversity panel (ADP). Trials were abbreviated based on the location, the year and the planting season (sequentially A to D). Harvested plot size can differ from the indicated planted plot size. The trials represent different growth conditions such as different nitrogen (N) and phosphorus (P) availability as well as irrigation, heat and drought.

Trial	Year	Location	GPS location	Elevation (masl)	Soil type	Plot length (m)	Plot width (m)	Plot rows (No.)	Replicate (No.)	Conditions	Lines (No.)
TZMo13D	2013	Morogoro, Tanzania	6°51’14.2”S 37°39’27.6”E	526	Ultisol	8.0	0.5	1	2	Low N, 2 mg P/kg	268
TZMb13D	2013	Mbeya, Tanzania	8°54’51.8”S 33°31’04.9”E	1780	Haplic Andosol	8.0	0.5	1	2	Low N	268
MZCH14D_drt	2014	Chokwe, Mozambique	24°30’03.6”S 33°00’09.2”E	35	n.a.	5.0	1.2	2	3	Drought	120
TZMo14D	2014	Morogoro, Tanzania	6°51’14.2”S 37°39’27.6”E	526	Ultisol	8.0	0.5	1	2	10 mg P/kg	192
TZMb14D	2014	Mbeya, Tanzania	8°54’51.8”S 33°31’04.9”E	1780	Haplic Andosol	8.0	0.5	1	2	Low N, 5 mg P/kg	192
TZAr14D	2014	Arusha, Tanzania	3°21’40.7”S 36°37’34.3”E	1387	Nitisol	8.0	0.5	1	2	21 mg P/kg	192
MZCH15A_heat	2015	Chokwe, Mozambique	24°30’03.6”S 33°00’09.2”E	35	n.a.	4.0	1.2	2	3	Heat	120
MZCH15C_irr	2015	Chokwe, Mozambique	24°30’03.6”S 33°00’09.2”E	35	n.a.	4.0	1.2	2	3	Irrigated	120
TZMb15D	2015	Mbeya, Tanzania	8°54’51.8”S 33°31’04.9”E	1780	Haplic Andosol	5.0	0.5	4	3	12 mg P/kg	41
TZAr15D	2015	Arusha, Tanzania	3°21’40.7”S 36°37’34.3”E	1387	Nitisol	5.0	0.5	4	3	Low N, 11 mg P/kg	41
ARBC16D_irr	2016	Wilcox, Arizona	32°01’43.9”N 109°41’27.3”W	1321	Entisol	3.0	1.0	2	3	Irrigated	110

Supplementary Table 4. Description of the field sites and trials from already published data. This data from the elite Andean breeding panel (VEF), the Mesoamerican introgression panel (MIP), Andean x Mesoamerican panel (AxM) was included in the analysis of this study. Trials were abbreviated based on the location, the year and the planting season (sequentially A to D). The trials represent different growth conditions such as different phosphorus (P) availability as well as irrigation, heat and drought.

Trial	Year	Location	Condition	Panel	Lines	Data source
Dar16C_hiP	2016	Darién, Colombia	High P	VEF	357	Keller et al. (2020)
Dar16C_loP	2016	Darién, Colombia	Low P	VEF	360	Keller et al. (2020)
Dar16C_mdP	2016	Darién, Colombia	Medium P	VEF	360	Keller et al. (2020)
Pal13C_drt	2013	Palmira, Colombia	Drought	VEF	159	Keller et al. (2020)
Pal15C_drt	2015	Palmira, Colombia	Drought	VEF	356	Keller et al. (2020)
Pal15C_irr	2015	Palmira, Colombia	Irrigated	VEF	357	Keller et al. (2020)
Pal16C_drt	2016	Palmira, Colombia	Drought	VEF	357	Keller et al. (2020)
Pal17C_drt	2017	Palmira, Colombia	Drought	VEF	318	Keller et al. (2020)
Pal18A_irr	2018	Palmira, Colombia	Irrigated	VEF	319	Keller et al. (2020)
Pal18C_drt	2018	Palmira, Colombia	Drought	VEF	276	Keller et al. (2020)
Pal18A_irr	2018	Palmira, Colombia	Irrigated	MIP	217	Diaz et al. (2021)
Pal13C_drt_AxM	2013	Palmira, Colombia	Irrigated	AxM	200	Mayor Duran et al. (2016)
Pal15A_irr_AxM	2015	Palmira, Colombia	Irrigated	AxM	200	Mayor Duran et al. (2016)
Pal13C_irr_AxM	2013	Palmira, Colombia	Irrigated	AxM	200	Mayor Duran et al. (2016)

Supplementary Table 5. Variance components for the lines of the climbing bean panel (VEC) and genotype x environment interaction between lines and trials for each trait. The basic model (1, see Material and Methods) was compared to a model with an interaction effect (Line:trial; Model GxE). The traits 100 seed weight (100SdW), days to flowering (DF), days to physiological maturity (DPM), seed iron concentration (SdFe) and seed yield were evaluated.

Effect	Component	Trait	Model
Line	36.30	100SdW	(1)
Line	29.82	100SdW	GxE
Line:trial	18.22	100SdW	GxE
Line	7.52	DF	(1)
Line	6.59	DF	GxE
Line:trial	2.84	DF	GxE
Line	13.86	DPM	(1)
Line	11.19	DPM	GxE
Line:trial	4.22	DPM	GxE
Line	90.05	SdFe	(1)
Line	85.17	SdFe	GxE
Line:trial	10.56	SdFe	GxE
Line	30394.70	Yield	(1)
Line	23099.87	Yield	GxE
Line:trial	94971.58	Yield	GxE

Supplementary Table 6. Significant marker-trait associations within the climbing bean panel (VEC) below the 5% significance level as revealed by genome-wide association studies. The trait, chromosome (Chr), physical position (Pos), association strength (p value), name of the marker and minor allele frequency (MAF) of the significantly associated SNPs is reported. Significant associations were identified for canning quality, days to flowering (DF), days to physiological maturity (DPM), pod harvest index (PHI), seed iron concentration (SdFe) and seed iron zinc (SdZn) based on 290 lines.

	Trait	Chr	Pos	p value	Marker	MAF
1	Canning	Chr 7	2671540	2.54e-06	Chr07_2671540_8858	0.36
2	DF	Chr 1	41082650	1.38e-06	Chr01_41082650_841	0.08
3	DF	Chr 4	7150323	5.71e-07	Chr04_7150323_5543	0.14
4	DF	Chr 5	9116341	7.29e-11	Chr05_9116341_6608	0.09
5	DF	Chr 5	26823068	5.15e-10	Chr05_26823068_6755	0.06
6	DF	Chr 5	39344071	7.48e-11	Chr05_39344071_7131	0.07
7	DF	Chr 11	1341682	9.12e-07	Chr11_1341682_14330	0.25
8	DPM	Chr 6	20010344	8.8e-07	Chr06_20010344_7757	0.09
9	DPM	Chr 8	21158707	3.48e-07	Chr08_21158707_10677	0.47
10	PHI	Chr 5	38677400	7.74e-21	Chr05_38677400_7053	0.34
11	PHI	Chr 6	19939926	1.62e-06	Chr06_19939926_7748	0.12
12	PHI	Chr 7	4874521	1.04e-07	Chr07_4874521_9066	0.10
13	PHI	Chr 10	42780526	5.11e-15	Chr10_42780526_14008	0.10
14	SdFe	Chr 2	2906306	1.04e-07	Chr02_2906306_1914	0.24
15	SdFe	Chr 2	46566153	2.41e-06	Chr02_46566153_3097	0.14
16	SdFe	Chr 2	48864009	2.7e-09	Chr02_48864009_3327	0.13
17	SdFe	Chr 4	46178140	3.58e-08	Chr04_46178140_5942	0.24
18	SdFe	Chr 6	26892426	8.02e-09	Chr06_26892426_8218	0.08
19	SdFe	Chr 7	4952125	2.13e-10	Chr07_4952125_9069	0.09
20	SdFe	Chr 10	44211585	7.99e-10	Chr10_44211585_14161	0.09
21	SdZn	Chr 2	2552253	4.17e-09	Chr02_2552253_1890	0.41
22	SdZn	Chr 10	7067350	3.77e-11	Chr10_7067350_13396	0.14

Supplementary Table 7. Highest achieved prediction accuracies (MaxPAcc) for new lines of the climbing bean panel (VEC) among all models for each trial and across all trials. The traits 100 seed weight (100SdW), days to flowering (DF), seed iron concentration (SdFe) and seed yield were evaluated. Prediction accuracy was defined as the quotient of prediction ability and the square root of heritability. Trials were abbreviated based on the location Darién (Dar), Palmira (Pal), Popayán (Pop) in Colombia, Kagera in Tanzania (TzKg), or Kawanda in Uganda (UgKw), the year and the planting season (sequentially A to D). For a detailed description of each trial see Supplementary Table 2.

	Trait	Trial	maxPAcc	Model
1	100SdW	Dar14B	0.77	Genotype (among trials)
2	100SdW	Dar18B	0.67	Genotype (single trials)
3	100SdW	Dar19B	0.69	Genotype (single trials)
4	100SdW	Pop15B	0.69	GxE
5	100SdW	Pop17D	0.68	Genotype (single trials)
6	100SdW	Pal19D	0.67	Genotype (single trials)
7	100SdW	TzKg19D	0.49	Genotype (single trials)
8	100SdW	BLUEs	0.80	TP VEC
9	DF	Dar14B	0.53	Factor analysis
10	DF	Dar18B	0.67	Factor analysis
11	DF	Dar19B	0.57	Factor analysis
12	DF	Pop15B	0.61	Genotype (among trials)
13	DF	Pop17D	0.52	Factor analysis
14	DF	Pal19D	0.53	Factor analysis
15	DF	UgKw19D	0.53	Factor analysis
16	DF	BLUEs	0.67	TP optimized
17	SdFe	Dar14B	0.42	Genotype (single trials)
18	SdFe	Dar18B	0.56	Genotype (single trials)
19	SdFe	Dar19B	0.56	Genotype (among trials)
20	SdFe	Pop17D	0.64	Genotype (single trials)
21	SdFe	BLUEs	0.67	TP optimized
22	Yield	Dar14B	0.37	Genotype (single trials)
23	Yield	Dar18B	0.73	Genotype (single trials)
24	Yield	Dar19B	0.62	Genotype (single trials)
25	Yield	Pop15B	0.25	Genotype (single trials)
26	Yield	Pop17D	0.70	Genotype (single trials)
27	Yield	Pal19D	0.33	Genotype (single trials)
28	Yield	TzKg19D	0.42	Genotype (single trials)
29	Yield	UgKw19D	0.50	Genotype (single trials)
30	Yield	BLUEs	0.23	TP optimized
